# *Staphylococcus aureus* induced trained immunity in macrophages confers heterologous protection against gram-negative bacterial infection

**DOI:** 10.1016/j.isci.2024.111284

**Published:** 2024-10-29

**Authors:** Simon R. Carlile, Seán C. Cahill, Eóin C. O’Brien, Nuno G.B. Neto, Michael G. Monaghan, Rachel M. McLoughlin

**Affiliations:** 1 Host-Pathogen Interactions Group, School of Biochemistry and Immunology, Trinity Biomedical Sciences Institute, Trinity College Dublin, Dublin, Ireland; 2 Department of Mechanical, Manufacturing and Biomedical Engineering, Trinity College Dublin, Dublin, Ireland

**Keywords:** Immunology, Microbiology

## Abstract

*Staphylococcus aureus* can induce trained immunity in murine macrophages offering protection against repeat exposure during *S. aureus* skin infection. Here we demonstrate that *S. aureus* exposure can result in non-specific trained immunity in humans and mice, enhancing macrophage responsiveness and bacterial clearance in a heterologous challenge. In humans, the enhanced macrophage responsiveness was accompanied by metabolic changes and histone modification. In mice, the enhanced responsiveness of macrophages occurred in conjunction with enhanced myelopoiesis. This report provides further insights on the host’s response to the bacterium *S. aureus*, indicating that exposure to this organism induces heterologous protection against subsequent gram-negative infection that is provided by macrophages. These findings support the hypothesis that *S. aureus* has evolved to develop a mutualistic relationship with the host, imbuing the host with enhanced capacity to protect itself from attack by alternative pathogens, while potentially allowing *S. aureus* to exert its dominance within its niche.

## Highlights

• *S. aureus* can induce non-specific trained responses from human macrophages
• Systemic *S. aureus* exposure can alter progenitor cell populations in the bone marrow of mice
• Systemic exposure to *S. aureus* provides protection against systemic *E. coli* challenge

## Graphical abstract

**Figure:**
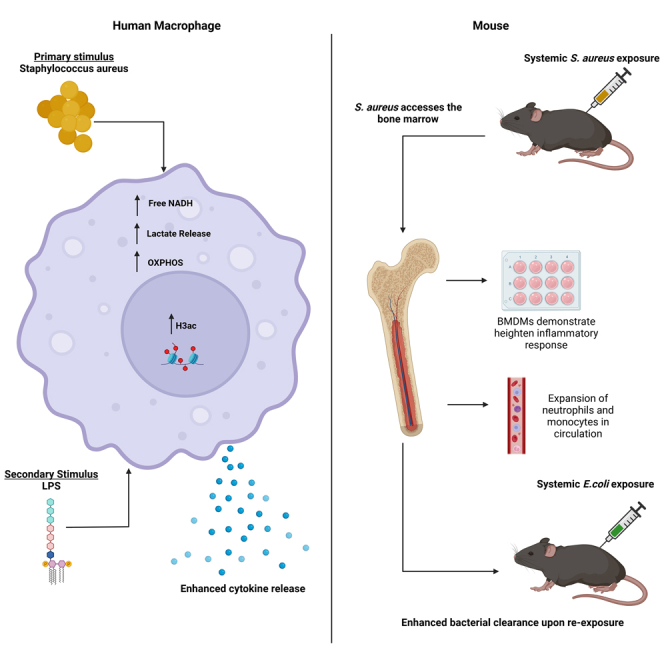

## Introduction

*Staphylococcus aureus* is traditionally viewed as an opportunistic pathogen and is a leading cause of skin and soft tissue infection and bacteraemia. In contrast, *S. aureus* is also part of the normal human microbiome.^1^ The evolution of *S. aureus* as an almost ubiquitous colonizer of humans, raises the question that it may be more than just an opportunistic pathogen and indicates that a highly adapted relationship is maintained between this organism and the host’s immune system.^2^ Immunological memory has evolved to ensure survival when an organism is exposed to a previously encountered pathogen. However heterologous immunity, which is defined as immunity that can be induced by one pathogen against another unrelated pathogen,^3^ explains how the host has evolved to resist the enormous pressures exerted by the diversity of microbes that are encountered. It also explains why certain species of bacteria have evolved to dominate as colonizers of specific hosts. An individual’s response to a pathogen will be significantly influenced by their prior microbial encounters both in the context of exposure to pathogens and commensal microbes.^4^^,^^5^ Given *S. aureus*’ propensity for colonizing and infecting the human host, it stands to reason that immunological responses to other pathogens could be shaped by prior *S. aureus* exposure. To facilitate its success as a colonizer, it is possible that *S. aureus* has evolved mechanisms to confer protection against the influx of alternative pathogens, through immunomodulation that would ensure its dominance within its niche, while also supporting survival of its host.

There is a growing appreciation that myeloid cells, such as monocytes/macrophages can develop a memory like phenotype termed trained immunity and can contribute significantly to heterologous immunity. This “trained” phenotype has been shown to develop upon exposure of monocytes/macrophages to microbial derived compounds such as the Bacille Calmette-Guerin (BCG) vaccine and the fungal cell wall component β-glucan.^6^ As a result of these interactions, the monocytes/macrophages undergo metabolic and epigenetic reprogramming, which in turn leads to enhanced effector functions upon secondary exposure to a related or an unrelated microbe.^7^ Trained immunity can develop in peripheral tissues or can develop as a result of modifications to myeloid progenitor cell populations within the bone marrow, which explains why the trained phenotype can be maintained in myeloid cells over extended periods.^8^^,^^9^

*S. aureus* has been shown to generate a trained response in murine models of skin infection, with mice previously exposed to *S. aureus* demonstrating an enhanced ability to clear subsequent *S. aureus* skin challenges. This response was independent of the activity of T or B cells^10^ and relied upon improved phagocytic activity of macrophages.^11^ More recently it has been shown, in the skin of mice, that *S. aureus* can induce trained responses in CD64^Hi^ dermal macrophages, which drove a CXCL9 response upon rechallenge leading to enhanced neutrophil recruitment and had a direct antimicrobial effect.^12^ The importance of innate immune training in conferring protection against *S. aureus* is further evidenced by the fact that the bacterium has evolved mechanisms to circumvent it. Work by Lung et al. demonstrated that *S. aureus*’ adoption of a small colony variant phenotype can induce metabolic reprogramming of the host cell that impedes the induction of trained immunity in macrophages to facilitate its own persistence.^13^ These reports highlight the potential beneficial effect of prior *S. aureus* exposure in protecting against reinfection through the induction of a “trained-like” response at least in the mouse. Whether this translates to the human setting is an open question. Furthermore, it remains to be established if *S. aureus* induced reprogramming of myeloid cells is sufficient to confer heterologous protection, which would be consistent with bona fide trained innate immunity.

Here we demonstrate that *S. aureus* can induce a trained response in human macrophages, driven by metabolic changes and histone modifications that lead to enhanced effector functions in response to lypopolysaccharide (LPS) and *Escherichia* *coli.* Further, we demonstrate that systemic exposure to *S. aureus* induces a non-specific trained immune response in mice, enhancing inflammatory responses that promote bacterial clearance upon subsequent systemic *E. coli* infection. These protective effects are conferred via the induction of central trained immunity whereby *S. aureus* reprograms myelopoietic stem cells leading to enhanced monocyte and macrophage inflammatory responses, allowing the host to mount a more robust response upon *E. coli* challenge.

## Results

### *S. aureus* reprograms human peripheral blood derived macrophages toward enhanced effector functions

**Figure 1:**
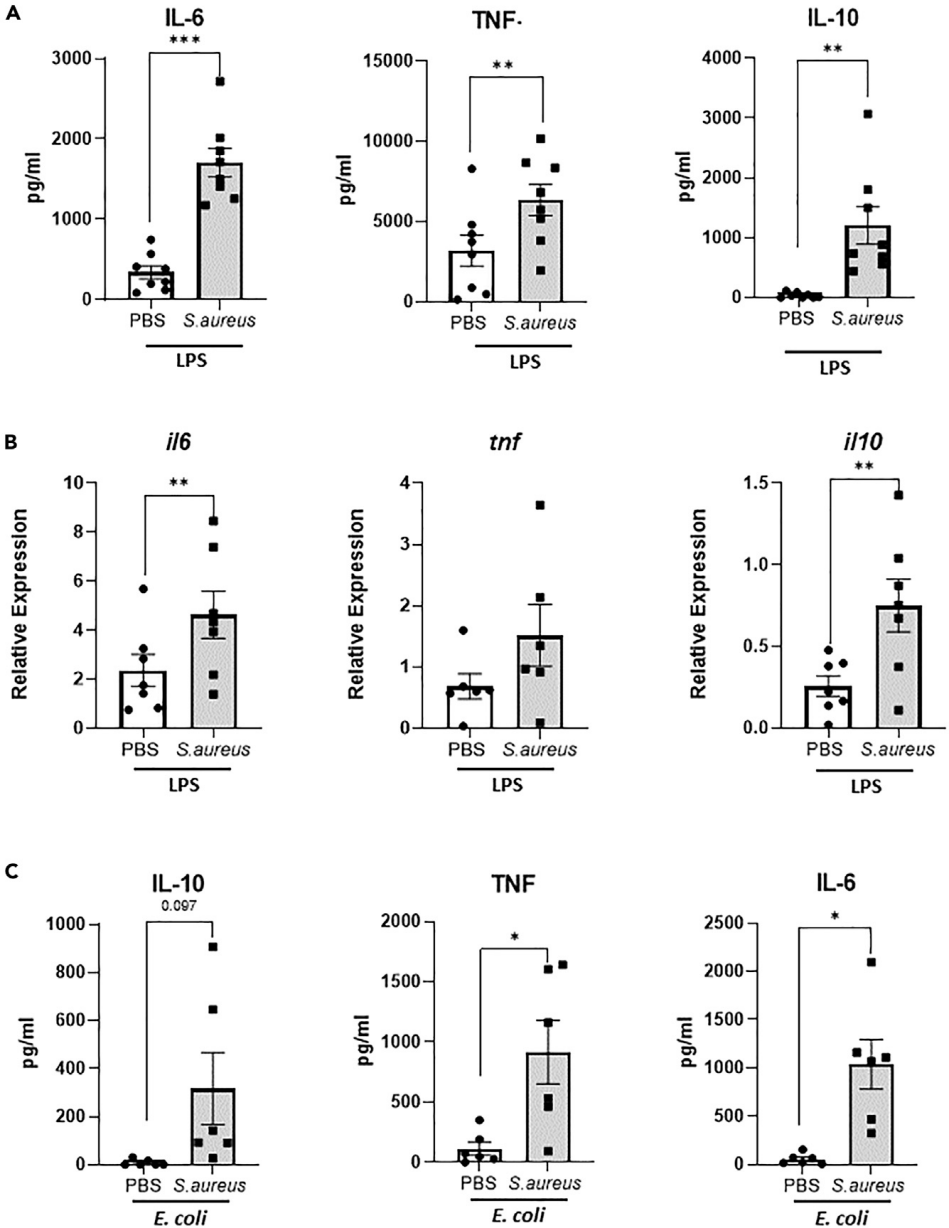
*S. aureus* exposure enhances human macrophage responses to subsequent LPS or *E. coli* challenge (A and B) Human peripheral blood monocytes were isolated by CD14^+^ selection and exposed to heat-killed *S. aureus* strain Newman (2 μg/ml) or PBS. Media was refreshed after 24 h and cells were maintained in RPMI supplemented with 10% pooled human serum. On day 7 macrophages were subsequently exposed to LPS and cytokine responses measured at the mRNA level at 3 h by RT-PCR (A) and protein level at 24 h by ELISA (B). (C) PBS and *S. aureus* exposed macrophages were also rechallenged with live *E. coli* (MOI100) and cytokine responses measured by ELISA at 24 h post challenge. Results expressed pg/ml or ΔΔct +/−SEM for *n* = 6–8 individual donors. Statistical significance measured by paired t test, *p* < 0.05 ∗, *p* < 0.01 ∗∗, *p* < 0.0001 ∗∗∗.

Trained immunity can be induced in human macrophages upon stimulation with microbial derived compounds such as β-glucan^6^ and the BCG vaccine.^14^ To investigate if *S. aureus* can similarly reprogram human macrophages in this way, peripheral blood derived monocytes were isolated from buffy coats from healthy donors and exposed to heat-killed *S. aureus* (strain Newman) *in vitro* for 24 h before resting for 7 days at which point, they were re-stimulated with LPS (10 ng/ml). Macrophages exposed to *S. aureus* responded to subsequent LPS stimulation with significantly enhanced interleukin (IL)-6, tumor necrosis factor (TNF), and IL-10 responses compared to control cells that had previously been exposed to PBS, as measured at the RNA level at 3 hr (Figure 1A) and protein level at 24 h (Figure 1B). Enhanced macrophage responses were also observed upon live *E. coli* challenge, with IL-6, TNF, and IL-10 production significantly increased in the cells that had previously been exposed to *S. aureus* as compared to control PBS exposed cells (Figure 1C). Prior exposure to *S. aureus* also enhanced the response of macrophages re-challenged with live *S. aureus* (Figure S1A). When macrophages were trained with live *S. aureus* or alternative strains of *S. aureus*, this also resulted in an enhanced secondary response to LPS (Figures S1B and S1C). There was no difference detected between PBS and *S. aureus* exposed macrophage on day 7 prior to rechallenge, indicating that macrophages return to a basal state and are not continuously expressing IL-6, TNF, and IL-10 (Figure S1D). Together these results indicate that exposure to *S. aureus* reprograms human macrophages toward enhanced cytokine responses upon secondary exposure to alternative pathogens, consistent with the induction of a trained response.

### *S. aureus* exposed macrophages demonstrate an increased rates of glycolysis and oxidative phosphorylation

**Figure 2:**
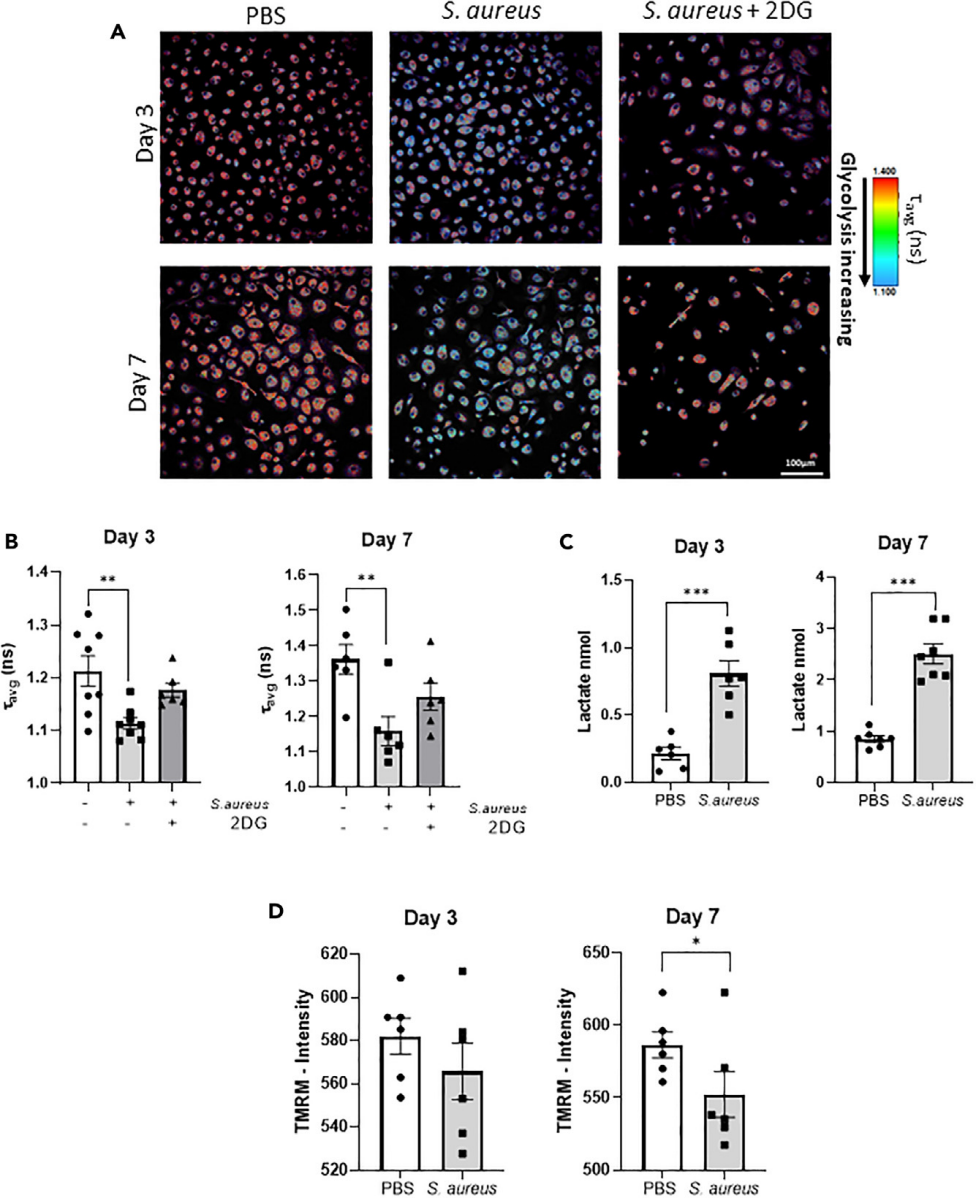
*S. aureus* trained macrophages increase basal glycolysis and oxidative phosphorylation Human peripheral blood monocytes were isolated by CD14^+^ selection and exposed to heat-killed *S. aureus* strain Newman (2 μg/ml) or PBS in presence or absence of 2DG. Media was refreshed after 24 h and cells were maintained in RPMI supplemented with 10% pooled human serum. On day 3 and day 7, 2P-FLIM analysis was used to measure the average fluorescence lifetime of NADH (τ-avg) as an indicator of the amount of bound or unbound NADH. (A) Representative image of FLIM analysis. (B) Average fluorescence lifetime (τ-avg) of NADH in PBS exposed compared *S. aureus* exposed macrophages on day 3 and day 7 was calculated. (C) Lactate release was measured in supernatants of *S. aureus* and PBS exposed macrophages on day 3 and day 7 by colorimetric assay. (D) Macrophages were stained with 250 nM TMRM for 1 h at 37°C and mitochondrial membrane potential was measured by multiphoton microscopy on day 3 and day 7. Results expressed as τ-avg, gmol, TMRM intensity +/−SEM for *n* = 6 individual donors. Statistical significance measured by paired t test (C and D) or one-way ANOVA with a Sidak’s test for multiple comparisons (B), *p* < 0.05 ∗, *p* < 0.01 ∗∗, *p* < 0.0001 ∗∗∗.

Having shown that *S. aureus* could reprogram human monocytes toward enhanced inflammatory responses upon subsequent related and unrelated pathogenic stimulation, it was important to establish if this was accompanied by metabolic changes that would support this trained response. To investigate glycolysis in *S. aureus* trained macrophages, 2-photon fluorescence lifetime imaging microscopy (2P-FLIM) was employed to assess the amount of bound or unbound NADH within the cytoplasm of the macrophages as an indicator of the glycolytic rate of the cell.^15^ 2P-FLIM analysis revealed an increase in unbound NADH in *S. aureus* trained cells compared to PBS-treated macrophages on day 3 and day 7 post initial exposure to *S. aureus* (Figure 2A), this is reflected in the significantly lower average lifetime fluorescence (τ-avg) in the *S. aureus* trained macrophages (Figure 2B). A return almost to baseline was observed when macrophages were trained with *S. aureus* in the presence of the glycolytic inhibitor, 2-dexoxy-D-glucose (2DG). Concurrent with previous reports,^16^ the presence of 2DG during training, limited the secondary response to LPS as indicated by a reduction in cytokine release (Figure S2A). Lactate dehydrogenase (LDH) assay analysis indicates a limited cytotoxic effect exerted by 2DG on day 3 and day 7 (Figure S2B). Lactate release from macrophages was also measured as another indicator of glycolysis. *S. aureus* trained macrophages produced significantly elevated levels of lactate on day 3 and day 7 post training when compared to PBS treated macrophages (Figure 2C). These data indicate that exposure to *S. aureus* induces a prolonged increase in the basal glycolytic rate of macrophages that is consistent with the induction of a trained immunity phenotype.^17^ Trained macrophages are not only more glycolytic but also exhibit an increase in oxidative phosphorylation.^18^ We also observed a decrease in the mitochondrial membrane potential within *S. aureus* trained macrophages assessed by multiphoton microscopy (Figure 2D), indicating that an increase in oxidative phosphorylation accompanied the increase in glycolysis induced by *S. aureus*.

### *S. aureus* induced training of human macrophages is associated with histone modifications

**Figure 3:**
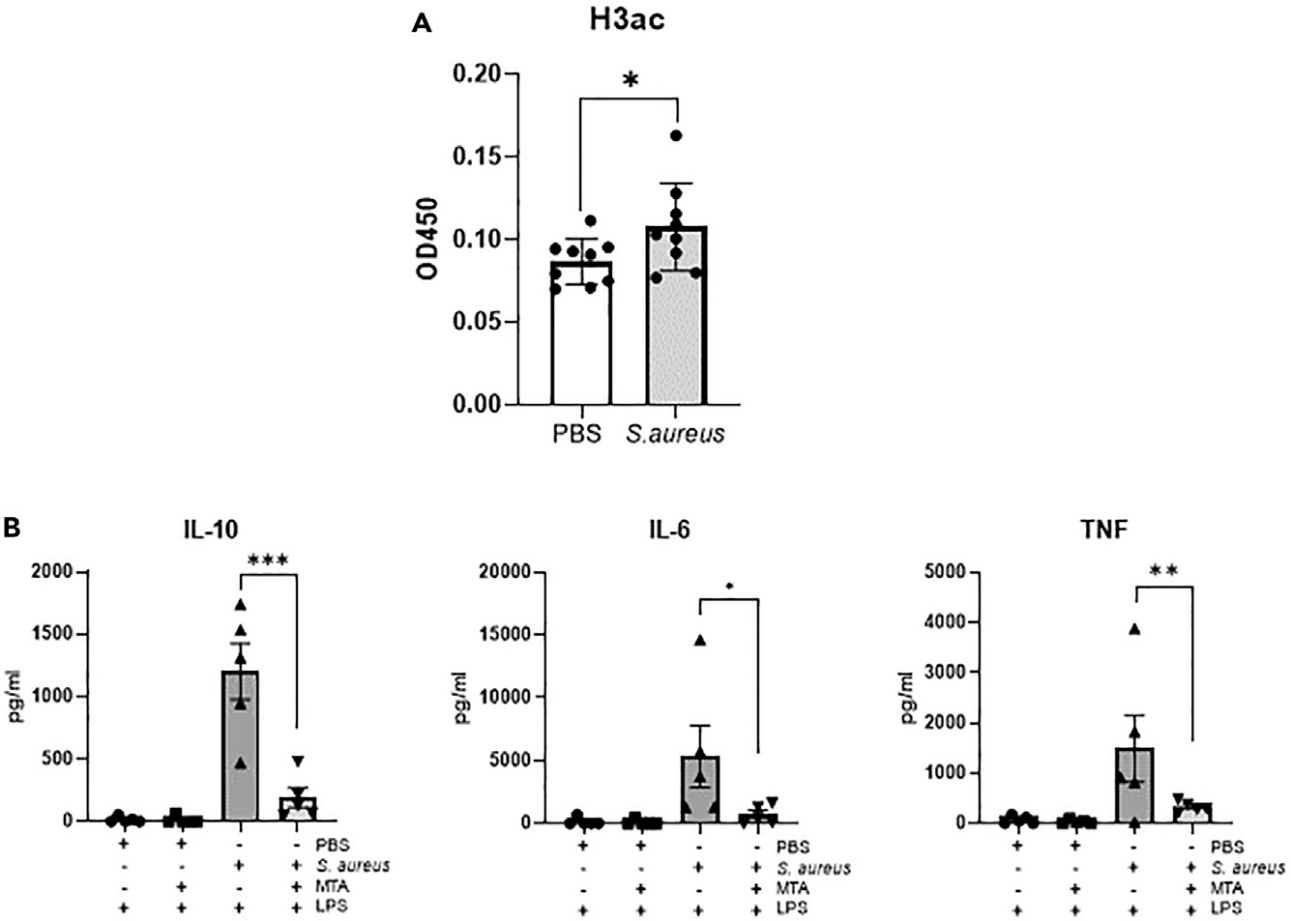
*S. aureus* exposure leads to histone modifications in macrophages that are required to support enhanced responses to subsequent LPS challenge (A and B) Human peripheral blood monocytes were isolated by CD14^+^ selection and exposed to heat-killed *S. aureus* strain Newman (2 μg/ml) or PBS. Macrophage lysates were collected on day 3 post training and sonicated to shear DNA. To assess histone 3 acetylation a pathscan ELISA on lysates was carried out (A). Monocytes were exposed to heat-killed *S. aureus* strain Newman (2 μg/ml) or PBS in the presence or absence of the methyltransferase inhibitor MTA (1 mM). Media containing MTA and the training stimulus was removed after 24 h and replaced with RPMI supplemented with 10% pooled human serum and stimulated with LPS (10 ng/ml) on day 7. IL-6, TNF, and IL-10 production was measured by ELISA (B). Results expressed as pg/ml or absorbance at 450 ± SEM for *n* = 5–9 individual donors. Statistical significance measured by paired t test, *p* < 0.05 ∗, *p* < 0.01 ∗∗.

Metabolic changes are required for the histone modifications that underpin the development of a trained immunity phenotype.^18^ To establish if histone modifications were associated with *S. aureus* training, macrophages were collected on day 3 post initial exposure to *S. aureus* and global histone 3 acetylation was assessed by ELISA, as an indicator of more open chromatin.^19^ *S. aureus* trained macrophages demonstrated an increase in global H3 acetylation when compared to PBS treated macrophages on day 3 post training (Figure 3A). Studies have demonstrated that methyltransferase inhibitor 5′-deoxy-5′-(methylthio)adenosine (MTA) can prevent the induction of a trained phenotype as MTA^6^^,^^14^ can prevent trimethylation of histone 3 lysine 4.^20^ We observed that the addition of the methyltransferase inhibitor MTA to monocytes 1 h prior to *S. aureus* exposure, significantly reduced the secondary response to LPS on day 7, with the levels of IL-6, TNF, and IL-10 significantly reduced in the cells that had been previously exposed to *S. aureus* in the presence of MTA compared to cells exposed to *S. aureus* alone (Figure 3B). These data indicate that *S. aureus* exposure leads to histone modifications in human monocyte/macrophages that create a more open chromatin structure, allowing for the enhanced response to secondary stimuli.

### Prior exposure to *S. aureus* confers protection against subsequent systemic *E. coli* infection *in vivo*

**Figure 4:**
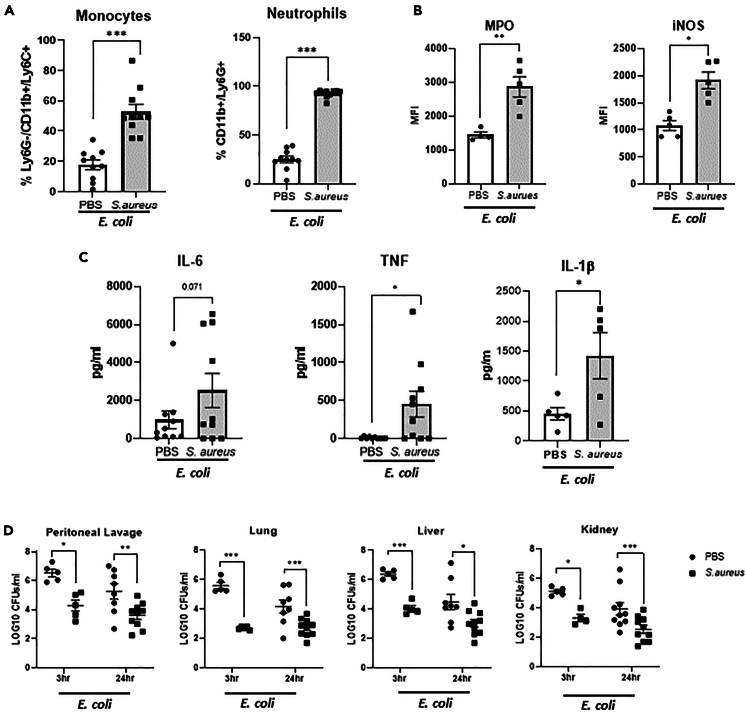
*S. aureus* exposure in mice offers protection upon subsequent *E. coli* challenge (A–D) Groups of C57/Bl6 mice were exposed to *S. aureus* through i.p. injection (5 × 10^8^ cfu/ml) on days 0, 7, and 14. Mice were allowed to rest for 21 days upon which mice are rechallenged i.p. with *E. coli* (1.5 × 10^7^ cfu/ml). Proportions of Ly6G + CD11b+ neutrophils and Ly6G-/CD11b+/Ly6C+ monocytes were analyzed by flow cytometry at 3 h post *E. coli* challenge (A) and expression of MPO and iNOS (MFI) on monocytes assessed by flow cytometry at 3 h post *E. coli* challenge (B). IL-6, TNF, and IL-1β levels in the peritoneal lavage fluid were quantified by ELISA at 3h post *E. coli* challenge (C). At 3 h and 24 h post *E. coli* challenge peritoneal cavity was lavaged and kidneys, lungs, livers were excised and homogenized. Lavage and homogenates were plated on TSA and allowed to grow overnight and total bacterial burden assessed (D). Results expressed as % of cells, mean fluorescence Intensity, pg/ml or LOG10CFU per mL +/−SEM for *n* = 4–10 individual mice. Statistical significance measured by unpaired t test (A–C) or two-way ANOVA with Sidak’s test for multiple comparisons (D), *p* < 0.05 ∗, *p* < 0.01 ∗∗, *p* < 0.0001 ∗∗∗.

Within the skin, prior exposure to *S. aureus* provided protection against subsequent *S. aureus* reinfection that was associated with enhanced macrophage functionality.^11^^,^^12^ However it is unknown if *S. aureus* exposure can similarly protect against subsequent infection with an alternative bacterium in a systemic setting. To investigate this, mice were exposed to *S. aureus* through multiple intraperitoneal (i.p.) challenges. On day 21 post the final exposure to *S. aureus,* by which time the *S. aureus* had been cleared^21^ (Figure S3A), mice were challenged with an i.p. injection of *E. coli*. Prior to *E. coli* challenge, there was no significant change in the number of resident neutrophil or monocyte populations present within in the peritoneal cavity of naive mice vs. those previously exposed to *S. aureus* (Figure S3B). At 3 h post *E. coli* challenge however, both the proportions (Figure 4A) and numbers (Figure S3C) of both cell populations were significantly elevated in the *S. aureus* exposed mice as compared to naive *E. coli* challenged mice (Figure 4A). Monocytes also displayed significantly increased expression of both iNOS and myeloperoxidase (MPO) (Figure 4B). There was also a trend toward an increase in the proportion of macrophages within the peritoneal cavity at 3 h post *E. coli* challenge (Figure S3D); however, the greatest changes were observed for neutrophils and monocytes. Associated flow cytometry gating strategy is shown in Figure S4. Significantly higher levels of TNF, IL-6, and IL-1β were also detected in the peritoneal lavage fluid in the *S. aureus* exposed mice compared to naive *E. coli* challenged mice (Figure 4C). Importantly, this enhanced local inflammatory response was associated with a reduction in the *E. coli* burden within the peritoneal cavity and also the systemic organs in the *S. aureus* exposed mice at 3 h and 24 h post *E. coli* challenge as compared to *E. coli* challenge of naive mice (Figure 4D). To confirm that this protection was primarily mediated by myeloid cells, CD3^+^ T cells were depleted using an anti-CD3 antibody prior to *E. coli* challenge (Figure S5A), which did not impact monocyte and neutrophil recruitment (Figure S5B) or clearance of *E. coli* at 3 h post *E. coli* challenge (Figure S5C). Together these data suggest an enhanced heterologous inflammatory response in mice that have previously been exposed to *S. aureus,* potentially due to the induction of trained immunity within myeloid cell populations that enhance monocyte effector functions and associated neutrophil recruitment.

### *S. aureus* exposure alters myeloid progenitor cell populations within the bone marrow

**Figure 5:**
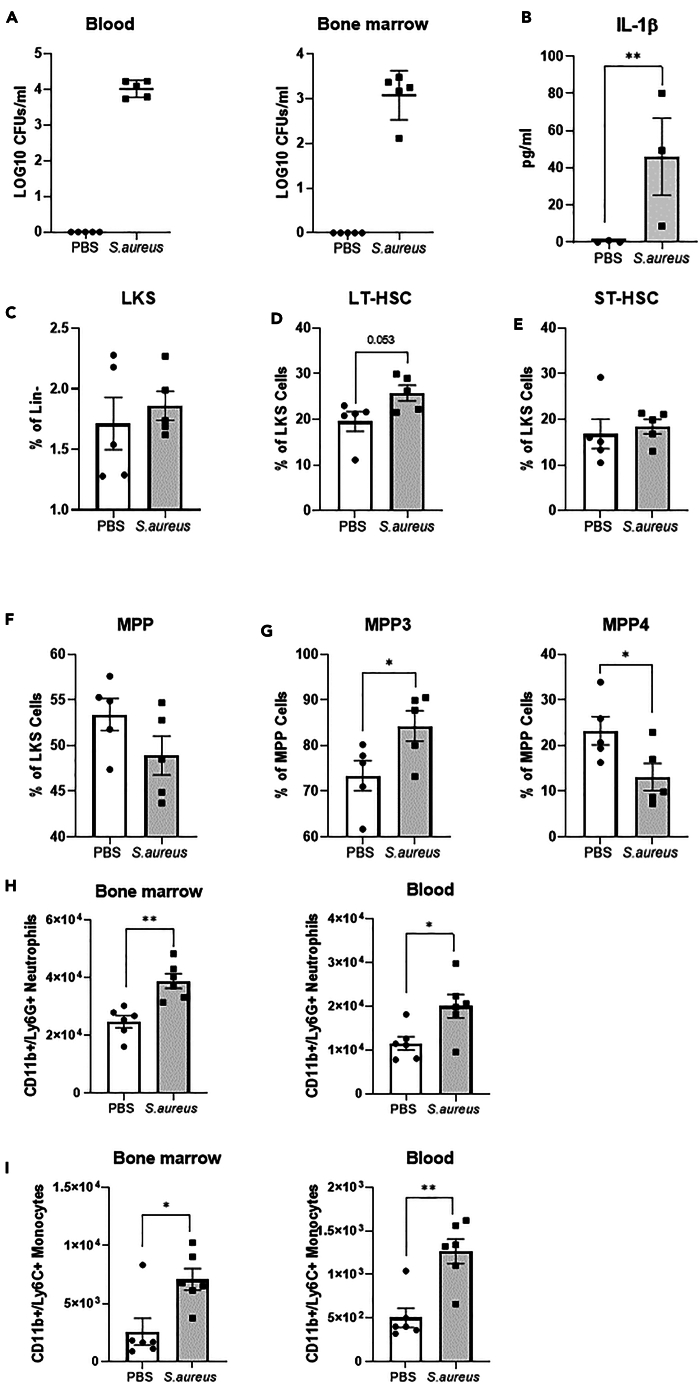
*S. aureus* exposure enhances myelopoiesis (A) Groups of C57Bl6 mice received a single i.p. challenge of *S. aureus* (5 × 10^8^ cfu). 3 h post challenge blood was collected and the left femur was excised. Femurs were flushed with 500 μl sterile PBS, cells were removed by centrifugation and 100 μl of the extracellular fluid and blood was plated on TSA and allowed to grow overnight and total bacterial burden assessed. (B) IL-1 β levels in bone marrow extra-cellular fluid was assessed by ELISA at 3 h post *S. aureus* challenge. (C–F) Groups of C57/Bl6 mice were exposed to *S. aureus* through i.p. injection (5 × 10^8^ cfu/ml) on days 0, 7, and 14. Mice were allowed to rest for 21 days upon which, bone marrow cells were isolated for phenotypic analysis by flow cytometry. Proportion of LKS cells (Lineage^-^cKit^+^Sca1^+^) (C), LT-HSCs (LKS^+^CD48^−^CD150^+^) (D), ST-HSCs (LKS^+^CD48^+^CD150^+^) (E), and MPP cells (LKS+CD48^+^CD150-) (F) in bone marrow were assessed. (G) Proportion of MPP3 (LKS+CD48^+^CD150-Flt3+) and MPP4 (LKS+CD48^+^CD150-Flt3-) cells were also assessed. (H and I) Neutrophil (H) and monocyte (I) numbers were assessed in bone marrow and in the blood on day 21 post final *S. aureus* or PBS exposure (*n* = 5–6). Results expressed as % of cells, absolute cell number, pg/ml or CFU per mL +/−SEM. Statistical significance measured by unpaired t test, *p* < 0.05 ∗, *p* < 0.01 ∗∗, *p* < 0.0001 ∗∗∗.

BCG and β-glucan can reprogram the myeloid progenitor cell populations in mice to induce long term trained immunity by enhancing myelopoiesis and the effector function of all subsequent myeloid cells.^8^^,^^9^ To explore this in *S. aureus* exposed mice, the capacity of *S. aureus* to access the bone marrow was assessed. At 3 h post i.p. challenge, *S. aureus* was detected in the blood and in the bone marrow (Figure 5A). This was associated with an increased expression of IL-1β within the bone marrow (Figure 5B), which has previously been implicated in the reprogramming of myeloid progenitor cells upon β-glucan challenge.^9^ To investigate if *S. aureus* exposure results in long term alternations to bone marrow progenitor populations, bone marrow cells were collected on day 21 post final exposure to *S. aureus* for phenotypic analysis by flow cytometry. Gating strategy is shown in Figure S6A. *S. aureus* exposure resulted in no significant change in the proportions of LKS cells (Lineage-cKit+Sca1+) (Figure 5C), short term hematopoietic stem cells (ST-HSCs, LKS+CD48^+^CD150+) (Figure 5E) or multipotent progenitor cells (MPP, LKS+CD48^+^CD150−); however, *S. aureus* exposure resulted in a trend toward an increase in long term hematopoietic stem cells (LT-HSCs, LKS+CD48^−^CD150+) (Figure 5D), as well as a slight decrease in the double-negative population (Figure S6B). Further, there was an increase in the proportion of MPP3 (LKS+CD48^+^CD150-Flt3+) cells concomitant with a significant reduction in the MPP4 (LKS+CD48^+^CD150-Flt3−) subset (Figure 5E). These changes were associated with an overall increase in mature neutrophil (Figure 5H) and monocyte (Figure 5I) populations in the bone marrow and also the circulation. Gating strategy is shown in Figure S7. Taken together, *S. aureus* exposure appears to enhance hematopoiesis skewing it toward myelopoiesis that leads to enhanced numbers of circulating mature myeloid cells.

### *S. aureus* exposure enhances macrophage effector functions

**Figure 6:**
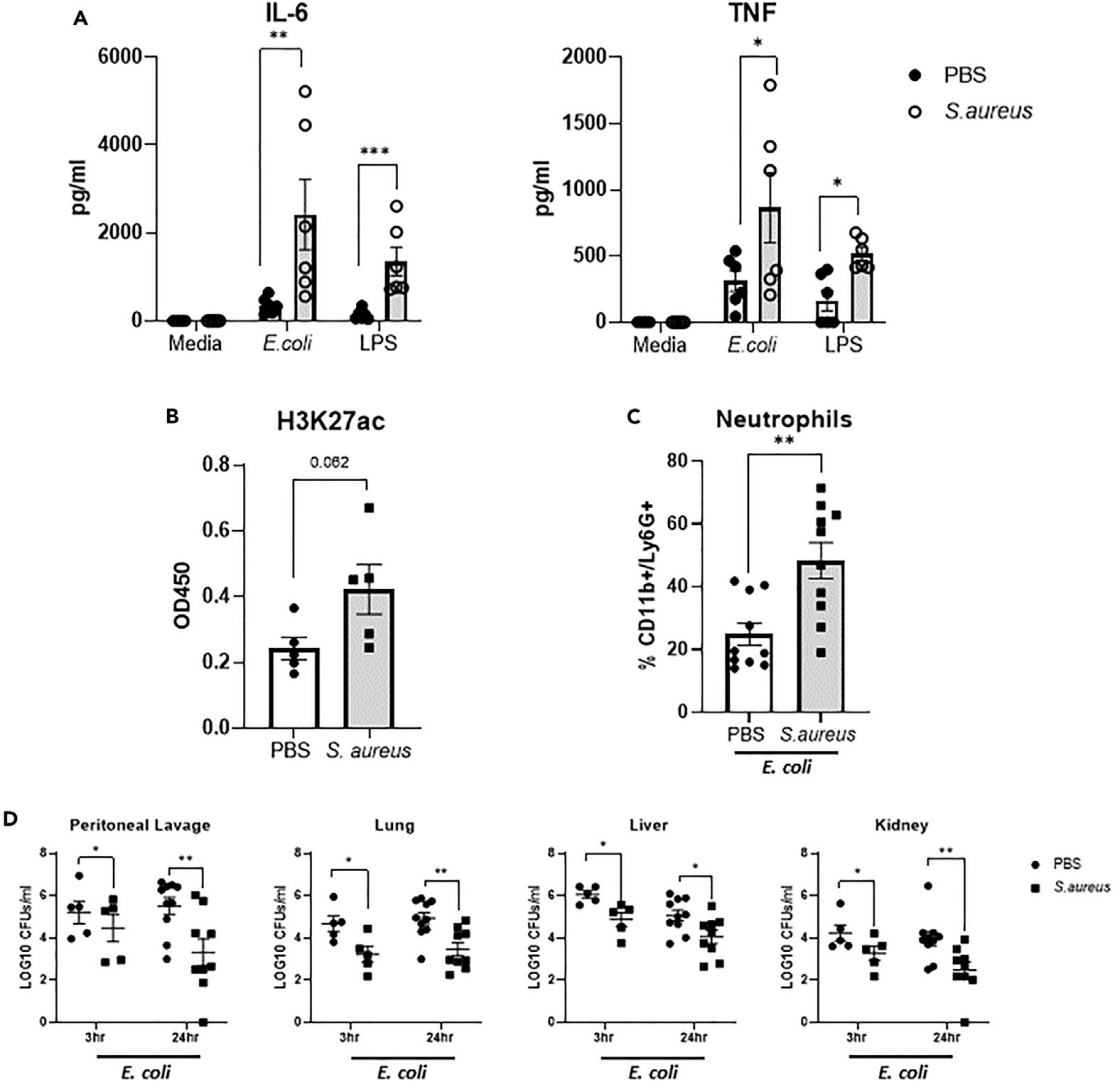
*S. aureus* exposure enhances macrophage effector functions Groups of C57/Bl6 mice were exposed to *S. aureus* or PBS through i.p. injection (5 × 10^8^ cfu/ml) on days 0, 7, and 14. Mice were allowed to rest for 21 days upon which BMDMs were generated and then challenged *in vitro* with LPS (10 ng/ml) or *E. coli* (MOI 100). (A–D) IL-6 and TNF production was measured by ELISA at 24 h (A). H3K27ac was assessed in BMDMs lysates from PBS and *S. aureus* exposed mice by ELISA (B). BMDMs from *S. aureus* exposed or PBS exposed mice were adoptively transferred into naive recipient mice 4 h prior to *E. coli* (1.5 × 10^7^ cfu) challenge. At 3 h post *E. coli* challenge, the peritoneal cavity was lavaged and the proportions of CD11b+/Ly6G + neutrophils analyzed by flow cytometry (C). At 3 h and 24 h post *E. coli* challenge, peritoneal lavage was collected and the lung, liver, and kidney were excised. Organs were homogenized and plated along with peritoneal lavage on TSA and allowed to grow overnight and total bacterial burden assessed (D). Results expressed as % of cells, pg/ml, absorbance at 450, LOG10CFU per mL for *n* = 6–10 individual mice, +/−SEM. Statistical significance measured by unpaired t test (A–C) or two-way ANOVA with Sidak’s test for multiple comparisons (D), *p* < 0.05 ∗, *p* < 0.01 ∗∗, *p* < 0.0001 ∗∗∗.

To establish if *S. aureus* exposure would lead to enhanced effector functions in myeloid populations, bone marrow-derived macrophages (BMDMs) were generated from PBS and *S. aureus* exposed mice on day 21 post final exposure to *S. aureus* and were stimulated with LPS or infected with *E. coli in vitro* prior to assessment of inflammatory cytokine production. TNF and IL-6 production were significantly enhanced in BMDMs generated from *S. aureus* exposed mice as compared to PBS-treated mice in response to both LPS and live *E. coli* (Figure 6A). Given this enhanced inflammatory response, expression of H3K27ac was assessed, as a marker of open chromatin, in BMDMs from PBS and *S. aureus* exposed mice, and was shown to be increased (Figure 6B) indicating the induction of histone modifications upon *S. aureus* exposure. Resident peritoneal macrophages isolated from PBS and *S. aureus* exposed mice on day 21 post final exposure to *S. aureus* also demonstrated an enhanced response to both LPS and *E. coli* stimulation (Figure S8). To confirm that centrally trained macrophages contributed to the heterologous immune protection seen in *S. aureus* exposed mice, BMDMs were generated from PBS and *S. aureus* exposed mice on day 21 post final exposure to *S. aureus* and adoptively transferred via i.p. injection into naive recipient mice, 4 h prior to *E. coli* challenge. At 3 h post *E. coli* challenge, mice receiving *S. aureus* trained BMDMs recruited significantly more neutrophils (Figure 6C) to the peritoneal cavity as compared to mice receiving BMDMs from PBS treated mice and this was associated with significantly reduced bacterial burden at the site of infection as well as systemically at 3 h and 24 h post *E. coli* challenge (Figure 6D). Interestingly, BMDMs isolated from *S. aureus* exposed mice did not exhibit an enhanced capacity to directly kill intracellular *E. coli* (Figure S9), suggesting that the protective effect is not due to improved phagocytic clearance but rather enhanced inflammatory activity of macrophages due to reprogramming of myeloid progenitor cells upon *S. aureus* exposure.

## Discussion

This study demonstrates the ability of *S. aureus* to induce non-specific trained immunity in mice and in human macrophages. Upon exposure to *S. aureus*, human monocytes exhibit the metabolic and epigenetic changes synonymous with trained immunity, demonstrating an increase in basal glycolysis and oxidative phosphorylation, and an increase in global histone 3 acetylation. These metabolic and epigenetic modifications were accompanied by an enhanced capacity to produce both pro (IL-6 and TNF) and anti (IL-10) inflammatory cytokines in response to subsequent challenge with LPS and *E. coli.* When histone methylation was inhibited, the induction of this trained phenotype was suppressed. These data suggested that *S. aureus* exposure has the potential to offer protection in the context of heterologous infection. Using a murine model of systemic exposure to *S. aureus* it was shown that *S. aureus* exposure enhanced protection against subsequent *E. coli* challenge, driven by enhanced monocyte function in conjunction with enhanced neutrophil recruitment, which together, facilitated a more rapid clearance of *E. coli*. This heterologous protective immunity appears to be underpinned by *S. aureus*’ ability to modulate hematopoiesis, with this study reporting that systemic exposure to *S. aureus* leads to enhanced myelopoiesis. Importantly, BMDMs generated from mice exposed to *S. aureus* had the capacity to protect against subsequent *E. coli* challenge following adoptive transfer to naive mice, through enhanced neutrophil recruitment.

Trained immunity is considered an evolutionarily conserved mechanism to boost immune responses in an effort to fight ongoing infection, combat cellular exhaustion and potentially prevent subsequent infections.^22^ Previous reports have demonstrated how *S. aureus* induced trained responses can offer protection against subsequent rechallenge in the skin through enhanced macrophage responses. This report, however, significantly advances our understanding of *S. aureus* induced trained immunity by demonstrating that *S. aureus* exposure can imbue the host with heterologous protection. Furthermore, it provides insights into *S. aureus* capacity to modulate hematopoiesis, with exposure to *S. aureus* leading to an increase in myelopoiesis and an associated enhancement of macrophage effector functions. *S. aureus* exposure did not increase the capacity of macrophages to directly kill intracellular *E. coli* but instead promoted IL-6, TNF, and IL-1β production by these cells, leading to enhanced recruitment of neutrophils to clear the infection. Previous studies by Feuerstein et al.^12^ demonstrated that in the context of *S. aureus* skin infection, protection against *S. aureus* rechallenge was dependent upon enhanced resident macrophage populations, but was not dependent on enhanced myelopoiesis or enhanced functionality of circulating monocyte populations. It is possible that in the context of a localized skin infection, *S. aureus* may not be able to access the bone marrow to enhance myelopoiesis that is required to cf. systemic protection. In this current study, *S. aureus* was rapidly detected within the blood and bone marrow following systemic challenge and this led to increases in IL-1β production within the bone marrow. IL-1R signaling has been shown to underpin β-glucan central trained immunity,^9^ leading to an enhancement of myelopoiesis that resulted in an increase in myeloid cells in the bone marrow. Similarly, *S. aureus* exposure led to an increase in the proportion of MPP3 cells in the bone marrow resulting in increases in mature monocytes and neutrophils in the bone marrow and in circulation.

Similar to the BCG vaccine and β-glucan, *S. aureus* appears to be inducing central trained immunity, potentially conferring a long-lived trained phenotype. Central trained immunity has the potential to impact multiple tissue sites, as resident macrophage populations are replaced from monocytes in circulation, which means overtime tissues can become seeded with “trained” resident macrophages. This results in a protective effect that is not only long lived, but one that could provide protection at various body sites. This central trained immunity driven by *S. aureus* appears to be similar to what has been observed in gut commensals that can enhance myelopoiesis to control infection with more pathogenic bacteria.^23^ Germ-free mice that were recolonized with caecal contents from specific pathogen-free (SPF) mice demonstrated enhanced myelopoiesis and protection against a systemic listeria challenge when compared to germ-free mice. This raises the intriguing possibility that *S. aureus* as a commensal could be enhancing myelopoiesis and macrophage effector functions in an effort to enhance the host immune responses against other bacteria that would serve to protect its colonization niche.

The development of T cell memory upon exposure to *S. aureus* in mice has previously been shown to enhance clearance upon subsequent re-exposure to *S. aureus*.^21^ Whether these T cells have the capacity to cf. bystander heterologous protection as has been described following exposure to other pathogens,^24^^,^^25^ remains to be established. It is likely that innate and adaptive immunity work in combination to cf. the beneficial immune memory that is generated by *S. aureus* exposure, but dissecting the mechanisms of this cross-talk is beyond the scope of the current study. Evidence from the literature indicates that enhanced innate immunity can promote T cell responses, in particular, macrophages can secrete IL-1β^26^ and IL-12^27^ during infection, both of which have been shown to be important in activating T cell responses during bacterial infection. Following *S. aureus* exposure there was an enhanced IL-1β response in *S. aureus* exposed mice which in turn could conceivably contribute to enhancing T cell responses. However, protection against *E. coli* challenge was maintained following CD3 blockade, supporting the proposed hypothesis that protection is myeloid based, with the enhanced myelopoiesis creating a larger pool of monocytes and neutrophils to recruit to the site of infection.

While *S. aureus* exposure in mice has been shown to offer protection upon re-exposure, this does not appear to be the case in human infection, as recurrent *S. aureus* infections are common,^28^ suggesting that *S. aureus* has the capacity to overcome the enhanced responses driven by trained immunity. Small colony variants of *S. aureus* have been shown to impair the induction of trained immunity. This is achieved via the induction of fumC expression that encodes an enzyme that breaks down local fumarate at the site of infection.^13^ Fumarate induces trained immunity by inhibiting lysine demethylases allowing for the epigenetic changes that enhance inflammatory responses.^29^ So it appears that *S. aureus* may have the potential to limit the effectiveness of trained immunity to facilitate its own survival put potentially capitalizes upon its ability to drive heterologous immunity to exert its dominance within the host.

Interestingly, an increase in production of the anti-inflammatory cytokine IL-10 was observed in *S. aureus* trained human macrophages in response to LPS, *E. coli*, and *S. aureus* challenge. Previously, it has been shown that *S. aureus* drives IL-10 responses during colonization to suppress T cell responses,^30^ and in chronic biofilm infection *S. aureus* drives an IL-10 response from myeloid cells via the induction of lactate, in both cases promoting *S. aureus* persistence.^31^ This may be a mechanism that *S. aureus* is utilizing to its advantage in order to effectively colonize or infect the host. It is conceivable that during asymptomatic colonization, *S. aureus* could induce trained immune response that is associated with enhanced IL-10 production allowing *S. aureus* to persistent while simultaneously equipping the host with the capacity for heterologous protection.

Non-specific trained immunity has been implicated in the excessive inflammation in conditions such as cardiovascular disease^32^ and rheumatoid arthritis.^33^ Further, a recent study has shown that recurrent exposure to influenza can lead to lung damage due to excessive inflammatory responses driven by macrophages.^34^ This suggests that *S. aureus* trained immunity has the potential to exacerbate existing inflammatory conditions or be a causative factor in the development of new conditions. Interestingly, *S. aureus* exposure has led to organ rejection in mice, which appeared to be driven via an excessive IL-6 response upon transplantation^35^ whether these effects are linked to the induction of a trained immune phenotype, however, remain to be established. This report shines new light on the complexity of the *S. aureus*/host interactions, highlights the need for further research to explore the true implications of *S. aureus* trained immunity on the host.

In summary, this study demonstrates the induction of non-specific *S. aureus* trained immunity in mice and in humans that enhances macrophage functionality. Further, revealing that *S. aureus* can induce central trained immunity, which increases myelopoiesis land leads to enhanced monocyte/macrophage effector functions. This offers protection against subsequent unrelated infection. Questions remain regarding the context in which training is induced, whether it is only during infection, or if trained immunity could be seen in healthy persistently colonized individuals. If this was the case, it would have significant implications for the host, supporting a hypothesis whereby *S. aureus* could utilize trained immunity as a mechanism to exert its dominance within its niche, protecting against foreign invasion by alternative microbes while simultaneously ensure the survivability of the host.

### Limitation of the study

This study does not explore the full breadth of cytokines that could be enhanced in response to *S. aureus*, instead focusing on a select number of key cytokines. Further this study does not comprehensively explore the role of T cell in the enhanced protective effect seen in mice. While the protective effect seen in mice is demonstrated to be independent on T cell at early time points, there remains the potential that T cell are playing a role in *E. coli* clearance, potentially through bystander activation of T cells. Macrophage depletion studies would be required to definitively prove the role for trained macrophages. A further, outstanding question remains the longevity of the protective effect seen in *S. aureus* exposed mice. While the protective effect seen when *S. aureus* trained macrophages were adoptively transferred into naive mice would likely be limited to the lifespan of the macrophages administered, the protective effect in *S. aureus* exposed mice may last longer as it appears to be underpinned by the enhanced myelopoiesis at the level of the bone marrow. Previous studies have shown the longevity of central trained immunity to extend in a range of 3–6 months.^36^

## Resource availability

### Lead contact

Further information and any requests should be directed to and will be fulfilled by the lead contact, Rachel M. McLoughlin (rachel.mcloughlin@tcd.ie).

### Materials availability

This study did not generate new unique reagents.

### Data and code availability

• Data reported in this paper will be shared by the lead contact upon request.
• This study did not generate any new original data and code
• Any additional information required to reanalyze the data reported in this paper is available from the lead contact upon request

## Acknowledgments

This study was funded by a Wellcome Investigator Award (202846/Z/16/Z) and a Science Foundation Ireland Investigator Award (15/IA/3041) to R.M.M. Thank you to the Irish Blood Transfusion Service for the provision of buffy coats to this research.

## Author contributions

Conceptualization, S.R.C. and R.M.M.; methodology, S.R.C., E.C.B., N.G.B.N., M.G.M., and R.M.M.; investigation, S.R.C., E.C.B., N.G.B.N., and S.C., writing – original draft, S.R.C. and R.M.M.; writing – review & editing, S.R.C. and R.M.M.; funding acquisition, R.M.M.; visualization, S.R.C., E.C.B., N.G.B.N., and S.C.C.; supervision, R.M.M.

## Declaration of interests

The authors declare no competing interests.

## STAR★Methods

### Key resources table

**Table:** 

REAGENT or RESOURCE	SOURCE	IDENTIFIER
**Antibodies**		

CD3 BV510	MSC	Cat# 100234; RRID:AB_2562555
Ly6G BV421	Invitrogen	Cat# 127641; RRID:AB_2925553
CD11b PerCP-Cy5.5	BD Biosciences Ltd	Cat# 45-0112-82; RRID:AB_953558
F4/80 APC	BD Biosciences Ltd	Cat# 17-4801-82; RRID:AB_2784648
Ly6C APC-eflour780	Invitrogen	Cat# 47-5932-82;RRID;AB_2573992
Inos PE	BD Biosciences Ltd	Cat# 12-5920-82; RRID:AB_2572642
MPO FITC	Abcam	Cat# ab90812; RRID:AB_2050025
Sca-1 PeCy7	Life Tech	Cat# 25-5981-82; RRID:AB_469669
CD127 BV711	BD Biosciences Ltd	Cat# 565490; RRID:AB_2732059
CD34 FITC	BD Biosciences Ltd	Cat# 560238; RRID:AB_1645242
CD48 PerCPef710	Life Tech	Cat# 46-0481-82; RRID:AB_10853483
CD150 PE	MSC	Cat# 115903; RRID:AB_313682
CD135 BV421	MSC	Cat# BL-135315; RRID:AB_2571919
cKit APC	MSC	Cat# 105812; RRID:AB_313221
CD16/32 AF700	BD Biosciences Ltd	Cat# 56-0161-82; RRID:AB_493994
CD45R APC-Cy7	MSC	Cat# 103223; RRID:AB_313006
CD3 APC-Cy7	BD Biosciences Ltd	Cat# 560590; RRID:AB_1727461
CD5 APC-Cy7	MSC	Cat# 100649; RRID:AB_2860587
CD8a APC-Cy7	BD Biosciences Ltd	Cat# 557654; RRID:AB_396769
Ter119 APC-Cy7	BD Biosciences Ltd	Cat# 560509; RRID:AB_1645230
CD45 FITC	BD Biosciences Ltd	Cat# 11045185; RRID:AB_465051
CD3 PE-Cy7	Biolegend	Cat# 45-0037-42; RRID:AB_1732057
CD11b APC	BD Biosciences Ltd	Cat# 17011282; RRID:AB_469343
Ly6CAPC-Cy7	Invitrogen	Cat# 48-5932-82; RRID:AB_2573503
Ly6G PecCP-eflour710	Invitrogen	Cat# 47-9668-82; RRID:AB_2573893
Anti-mouse CD3	Assay Genie	Cat# IVMB0059
Armenian Hamster IgG isotype control	Assay Genie	Cat# IVMB0183

**Bacterial and virus strains**		

*Staphyloccocus Aureus* - Newman	ATCC	25904
*Staphyloccocus Aureus* – PS80	ATCC	12600
*Escherichia coli –* CFT073	ATCC	700928

**Critical commercial assays**		

Human IL-6 and IL-10 ELISA	Biolegend	430501, 430601
Human TNF ELISA	Bio-Techne	DY210
Murine IL-6 Duoset ELISA	Bio-Techne	DY406
Murine IL-10 Duoset ELISA	Bio-Techne	DY417
Murine TNF Duoset ELISA	Bio-Techne	DY410
Human H3 acetylation ELISA	Cell Signaling Technology	7232
Murine H3K27ac ELISA	Cell Signaling Technology	93244

**Experimental models: organisms/strains**		

C57/BL6 Mice	Trinity College Dublin	–

**Software and algorithms**		

Prism Graphpad	Prism	–
FlowJo	Tree Star	–

**Other**		

BD Canto Flow Cytometer	BD Biosceinces	–
BD Fortessa Flow Cytometer	BD Biosceinces	–
Cytek Auroa Flow Cytomter	Cytek	–

### Experimental model and study participant details

#### Animal

##### *In vivo* exposure models

C57BL6J wild-type mice were bred in house at the Trinity College Dublin Comparative Medicine Unit. In all experiments, inclusion criteria were healthy animals aged 6–12 weeks. There were no exclusions. Experiments were typically carried out 2 times with 5 mice per group to establish the reproducibility of the results and to accommodate processing and analysis of material. To reduce bias, mice in all experiments were matched for sex and age. For each experiment mice were allocated to their treatment group by randomization within blocks (Nuisance variables: sex, cage location). Groups were allocated by the person performing the experiment. All animal experiments were conducted in accordance with the recommendations and guidelines of the health product regulatory authority, the competent authority in Ireland and in accordance with protocols approved by Trinity College Dublin Animal Research Ethics Committee. Mice received *S. aureus* strain PS80 5 × 10^8^ cfu via i.p injection (100μL) or PBS (Sigma) (100μL). This was repeated on day 7 and again on day 14. Mice were allowed to rest for 21 days up to day 35 to allow the infection to clear.^21^ On day 35, mice received 1.5 × 10^7^ cfu *E coli* via i.p injection. Mice were sacrificed at 3 h and 24 h post *E. coli* challenge. Peritoneal lavage was collected in 3mL PBS at 3 h post *E. coli* challenge for analysis of immune cell population by flow cytometry and cytokine release by ELISA. Peritoneal lavage, liver, lung and kidney were collected at 24h for analysis of bacterial burden.

##### Cell culture of bone marrow derived macrophages

BMDMs were generated from mice exposed to *S. aureus* or naive mice. Bone marrow cells were isolated from the legs of mice on Day 21 post final exposure to *S. aureus* and cultured in cDMEM (supplemented with 10% FBS (Sigma), 1% L-glut (Sigma) and 1% Penicillin/Streptomycin (Sigma)) supplemented with 20% L929. On day 6, BMDMs were enumerated and seeded onto 24 well plates at 1x10^6^ cells per well and allowed to rest overnight. On day 7, the media was replaced with antibiotic free cDMEM (Sigma) and BMDMs were challenged with PBS, *E. coli* (multiplicity of infection (MOI) 100), or LPS (10 ng/ml) (Sigma). BMDMs were challenged with LPS for 24h. BMDMs were challenged with *E. coli* for 1h, upon which BMDMs were treated with gentamicin (Sigma) (200 μg/ml). Gentamicin was removed after 1 h and BMDMs are maintained in cDMEM for a further 22 h.

For intracellular survival, BMDMs were seeded at a density of 1x10^6^ cells per well in a 12 well plate. BMDMs were infected at a MOI of 100 for 1 h before gentamicin treatment for a further 1 h (200 μg/mL). After this time, gentamicin containing media was removed and replaced with antibiotic free cDMEM. At 24h post challenge, cells were lysed with PBS containing Triton X-100 (0.1%) for 10mins and cells serially diluted, plated onto TSA plates and incubated at 37°C overnight for CFU enumeration.

BMDMs were lysed on day 6 for analysis of histone 3 acetylation details. Lysates were sonicated at 5°C using a Bioruptor Pico (Diagenode) for 10 cycles. Lysates were centrifugated at 14,000rpm and resultant supernatant was taken for fastscan ELISA (Cell signaling technology).

##### Human monocyte cell culture

Peripheral blood mononuclear cells (PBMCs) were isolated from anonymized buffy coats obtained from healthy blood donors at the Irish Blood Transfusion Service (Dublin, Ireland) as approved by the School of Biochemistry and Immunology Research Ethics Committee of Trinity College Dublin.

PBMCs were isolated by density gradient centrifugation using Ficoll (Lymphoprep). Monocytes were isolated from PBMCs by magnetic separation using a CD14 MACS kit (Miltenyi Biotec). Monocytes were seeded onto cell culture dishes and allowed to adhere overnight. Monocytes were exposed to heat-killed *S. aureus* strain Newman or PS80 (2 μg/ml) or PBS for 24h, upon which the stimulus was removed, and cells maintained in RPMI (Sigma) supplemented with 10% pooled human serum, 1% Penicillin/Streptomycin (Sigma), and 1% L-Glut (Sigma). On day 7 macrophages were stimulated with LPS (10 ng/ml) and cytokine production analyzed by ELISA at 24h. Macrophages were also rechallenged with *E. coli* (MOI100) or *S. aureus* (MOI100), for 1h. Macrophages were treated with gentamicin (200 μg/ml) for 1h to remove bacteria. Gentamicin was removed and the macrophages were maintained in cRPMI for a further 22hrs upon which supernatants were collected for analysis of cytokine production by ELISA.Where indicated 5′-Deoxy-5′-(methylthio)adenosine (MTA) (1mM) (Sigma) was added 1 h prior to the addition of the training stimulus (heat-killed *S. aureus*/PBS).

Monocytes/macrophages were lysed on day 3 and day 7 for analysis of histone 3 acetylation details. Lysates were sonicated at 5°C using a Bioruptor Pico (Diagenode) for 10 cycles. Lysates were centrifugated at 14,000rpm and resultant supernatant was taken for pathscan ELISA.

### Method details

#### Bacteria

*S. aureus* strain Newman and PS80 have been previously described.^37^ Strains were grown from frozen stocks on TSA at 37°C for 18h. All bacterial suspensions were prepared in sterile PBS and concentrations measured at an optical density of 600nm. CFUs were verified by plating serial dilutions of each inoculum onto TSA.

*E. coli* CFT073 is an urosepsis UPSE isolate.^38^ This strain was grown from frozen stocks on TSA at 37°C for 18h. Upon which, a single colony was selected and grown in 15mL tryptic soy broth for 3 h to a log phase. The bacterial suspension was centrifuged at 3600rpm for 10mins and resuspend in PBS. The concentrations were measured at an optical density of 600nm. CFUs were verified by plating serial dilutions of each inoculum onto TSA.

#### CFU quantification

For peritoneal lavage, serial dilutions of lavage fluid were performed in PBS. Dilutions were plated on MacConkey’s Agar and allowed to grow over night at 37°C, upon which, colony forming units were enumerated. For organs, samples were homogenized in 1mL PBS and serially diluted. Dilutions were plated on MacConkey’s Agar and allowed to grow over night at 37°C, upon which colony forming units were enumerated.

To assess if *S. aureus* could access the bone marrow, mice received *S. aureus* strain PS80 5x10^8^ cfus via i.p injection (100μL) or PBS (100μL). 3h post challenge, legs were collected from the mouse. Bone marrow cells were flushed with 1mL PBS. Cells were pelleted at 300g for 5mins and 100μL of extracellular fluid was collected for analysis of cytokine release by ELISA. Further extracellular fluid was plated on TSA and allowed to grow over night at 37°C.

#### Bone marrow cell collection

For bone marrow phenotyping, legs were collected on Day 21 post final exposure to *S. aureus* or PBS. Bone marrow cells were flushed out from the femur with 500ul PBS. Cells were pelleted by centrifugation and RBC lysis was carried out to remove red blood cells. Magnetic cell separation was used to isolate CD117+ cells (Mac Miltenyi). The purified cell population was stained for flow cytometric analysis.

#### T cell blocking

To assess if T cell were required for enhanced protection against *E. coli*, mice were administered 200μg of anti-CD3 antibody or isotype control via i.p injection on day 20 post final *S. aureus* or PBS delivery 24h post antibody administration mice were subjected to *E. coli* challenge as detailed above.

#### Adoptive transfer of bone marrow derived macrophages in naive recipient mice

BMDMs were generated from *S. aureus* exposed mice or PBS mice as detailed above. 2x10^5^ BMDMs were adoptively transferred into naive recipient mice, via i.p delivery, 4h prior to i.p *E. coli* (1.5x10^7^) challenge. Mice were sacrificed at 3 h and 24 h post *E. coli* challenge. Peritoneal lavage was collected at 3 h post *E. coli* challenge for flow cytometric analysis and ELISA. Peritoneal lavage, liver, lung and kidney were collected at 24h for analysis of bacterial burden as described above.

#### ELISA and LDH assay

ELISAs were used to analyze protein release in supernatants from cell cultures and peritoneal lavage fluid of mice and were carried out as per manufacturer’s instruction. Human IL-10, Human IL-6 (Biolegend) and Human TNF (R and D systems). Murine IL-6, TNF-α, and murine IL-1β (R and D systems). Histone 3 acetylation was measured by Pathscan H3ac ELISA (Cell Signaling Technology). Lactate dehydrogenase assay (Thermo) was carried out on cell supernatants as per manufacturer instructions.

#### RT-PCR

mRNA was isolated from macrophages using the Norgen RNA purification kit as per manufacturer’s instructions. Yields were quantified using a BMG LABTECH SPECTRO star Nano machine plate reader and its accompanying LV plate. High-Capacity cDNA reverse transcription kit (ThermoFisher) was used to convert mRNA (250ng) to complementary DNA as per manufacturer’s instructions. iTaq Sybr Green and a CFX96 Touch Real-Time qPCR Detection System (Bio-Rad) was used to quantify mRNA, as per manufacturer’s instructions. Primer pairs (Sigma) are listed in the table below.

**Table:** Table of Primer pairs

IL-6	Forward 5′-GCAGAAAAAGGCAAAGAAT Reverse 5′-CTACATTTGCCGAAGAGC
IL-10	Forward 5′-GCCTTTAATAAGCTCCAAGAG Reverse 5′-ATCTTCATTGTCATGYAGGC
TNF	Forward 5′-AGGCAGTCAGATCATCTTC Reverse 5′-TTATCTCTCAGGTCCACG
18s	Forward 5′-ATCGGGGATTGCAATTATTC Reverse 5′-CTCACTAAACCATCCAATCG

#### Multiphoton microscopy and 2P-FLIM

Human Monocytes we trained with heat-killed *S. aureus* or PBS, were indicated 2-Deoxy-D-glucose (1mM) was added 1 h prior to the training stimulus to inhibit glycolysis. Monocytes/Macrophages were analyzed on day 3 and day 7 post training by Multiphoton imaging and TMRM staining. Cells were stained using 250nM of TMRM and fluorescence emission was collected at a wavelength range of 580–638 nm after excitation with 860 nm. Acquired images were analyzed using CellProfiler with a custom built project pipeline.^39^ 2P-FLIM was carried out as previously detailed^15^ using a custom upright (Olympus BX61WI) laser multiphoton microscopy system equipped with a pulsed (80 MHz) titanium: sapphire laser (Chameleon Ultra, Coherent, USA), water-immersion 25× objective (Olympus, 1.05NA) and temperature-controlled stage at 37°C. Two photon excitation of NAD(P)H and FAD+ fluorescence was performed at the excitation wavelength of 760 and 800 nm, respectively. A 458/64 nm and 520/35 nm bandpass filter were used to isolate the NAD(P)H and FAD+ fluorescence emissions based on their emission spectrum.

#### Flow cytometry

Cells from the peritoneal lavage were pelleted at 300g for 5mins, upon which cells were enumerated and incubated with Brefeldin A (5 μg/ml) for 4h at 37°C. Cells were then washed in PBS and incubated in Fc block (αCD16/CD32) (ThermoFisher) before extracellular surface staining with fluorochrome-conjugated antibodies against CD3, Ly6G, CD11b, F4/80, and Ly6C. Cells were fixed and permeabilized, followed by intracellular staining with fluorochrome-conjugated antibodies against iNOS and MPO. For bone marrow phenotyping, bone marrow cells were collected from legs of mice and stained with Fixable Viability e506 (ebioscience) then stained with lineage negative antibodies against Ly6C, Ly6G, CD8a, TER119, CD5, B220, Sca-1, CD127, CD34, CD48, CD150, Flt3, cKit, CD16/32. For phenotyping of monocytes and neutrophils in the bone marrow and circulation, cells where collected from mice femurs and blood. Cells were stained with Fixable Viability e506 (ebioscience) then stained for CD45, CD3, CD11b, Ly6C and Ly6G. Fluorescence minus one sample were used as controls. Flow cytometric data were acquired with Cytek Aurora, BD Fortessa or BD Canto and analyzed using FlowJo software (Tree Star, Inc).

### Quantification and statistical analysis

#### Data analysis and statistical testing

Statistical analyses were performed using GraphPad Prism 8 software (GraphPad Software, La Jolla, CA). For human and murine studies, differences between groups were analyzed using a Student’s t test, Mann-Whitney test, or one-way anova or two-way ANOVA with sidak test used to assess significance between selected groups. A *p* value ≤ 0.05 was considered significant. Details of individual test performed can be found in the figure legends.
