# Supplementary material for: Staphylococcus aureus induced trained immunity in macrophages confers heterologous protection against gram-negative bacterial infection
Source: iScience. 2024 Oct 29;27(12):111284. doi: 10.1016/j.isci.2024.111284 (PMC11607596; doi:10.1016/j.isci.2024.111284)
Supplement: Document S1. Figures S1–S9 [file mmc1.pdf]

iScience, Volume *27*

Supplemental information

Staphylococcus aureus induced trained immunity in macrophages confers heterologous protection against gram-negative bacterial infection

Simon R. Carlile, Seán C. Cahill, Eóin C. O'Brien, Nuno G.B. Neto, Michael G. Monaghan, and Rachel M. McLoughlin

Figure S1. ***S. aureus*** **exposure enhances macrophage responds to S. aureus and LPS challenge and** ***S. aureus*** **trained cells do not become hyperinflammatory.**

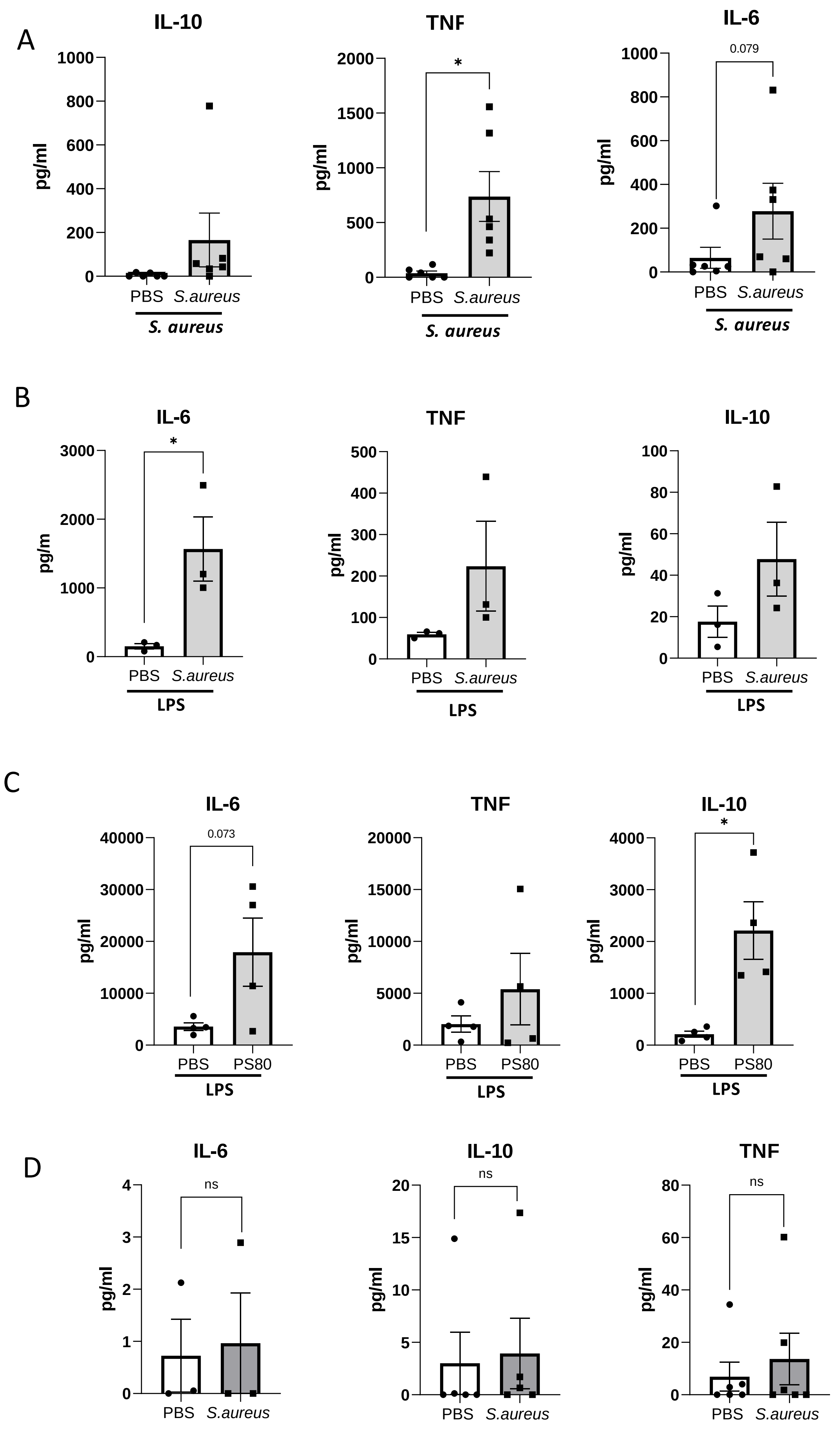

Figure S1*. S. aureus* exposure enhances macrophage responds to *S. aureus* and LPS challenge and *S. aureus* trained cells do not become hyperinflammatory.

(A) Human peripheral blood monocytes were isolated by CD14+ selection and exposed to heat-killed *S .aureus* strain Newman (2µg/ml) (A), live *S. aureus* strain Newman (MOI1) (B), heat-killed *S. aureus* strain PS80 (C) (2µg/ml) or PBS. Media was refreshed after 24hr and cells were maintained in RPMI supplemented with 10% pooled human serum. Macrophages were rechallenged with live *S. aureus* strain Newman (A) (MOI100) or LPS 10ng/ml (B+C). IL-6, TNF and IL-10 production at 24 hr was measured by ELISA. (D) On day 7 supernatants were collected from PBS and *S. aureus* treated cells prior to re stimulation and cytokines measured by ELISA. Results expressed as pg/ml +/-SEM for n=3-6 individual donors. Statistical significance measured by paired t-test, $p < 0.05$ *.

Figure S2. 2DG limits cytokine expression in *S.aureus* trained macrophages and does not significantly impact viability

A

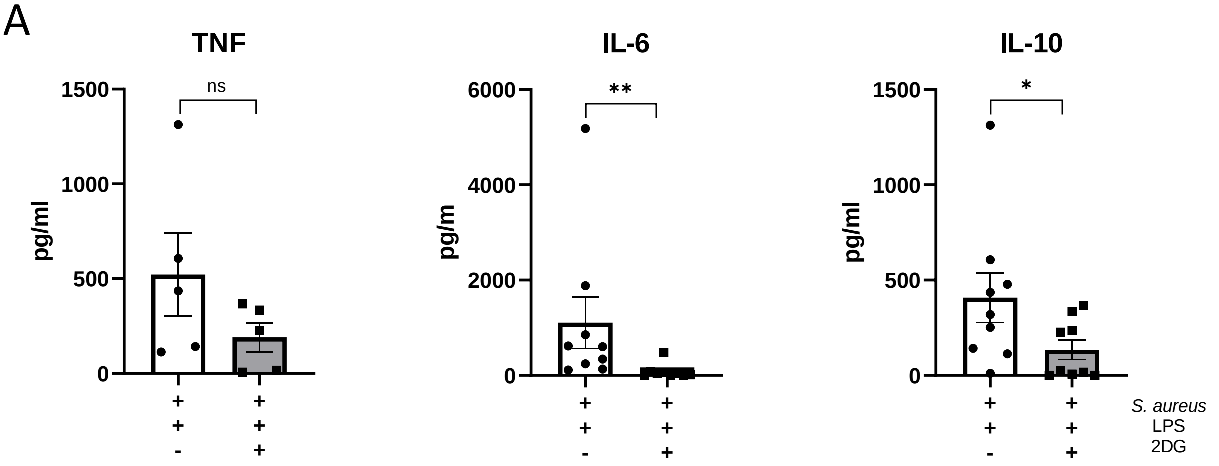

B

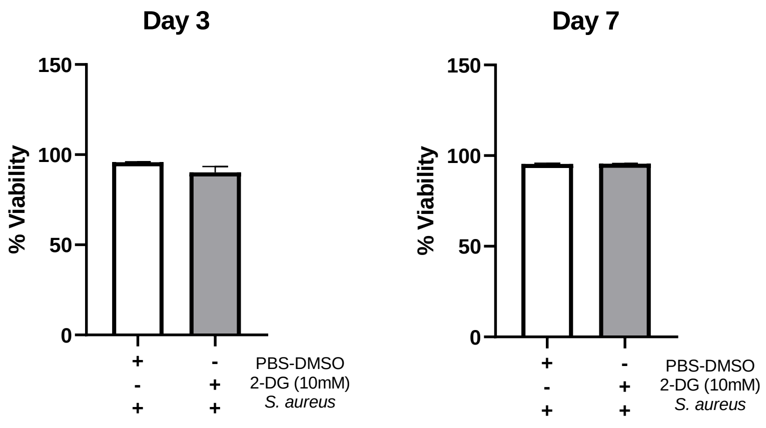

Figure S2. 2DG limits cytokine expression in *S. aureus* trained macrophages and does not significantly impact viability

Human peripheral blood monocytes were isolated by CD14+ selection. Monocytes were exposed to heat-killed *S. aureus* strain Newman (2μg/ml) or PBS in the presence or absence of 2DG (10mM). Media containing 2DG and the training stimulus was removed after 24hr. Cells were maintained in RPMI supplemented with 10% pooled human serum and stimulated with LPS (10ng/ml) on day 7. IL-6, TNF, and IL-10 production was measured by ELISA (A). Viability was assessed by lactate dehydrogenase assay on day 3 and day 7 prior to LPS challenge (B). Results expressed as pg/ml or % vaibility +/-SEM for n=5-8 individual donors. Statistical significance measured by mann whitney u test $p < 0.05$ *, $p < 0.01$ **.

Figure S3. Resident monocyte and neutrophil populations are not elevated in the peritoneal cavity at day 21 post exposure to *S. aureus*.

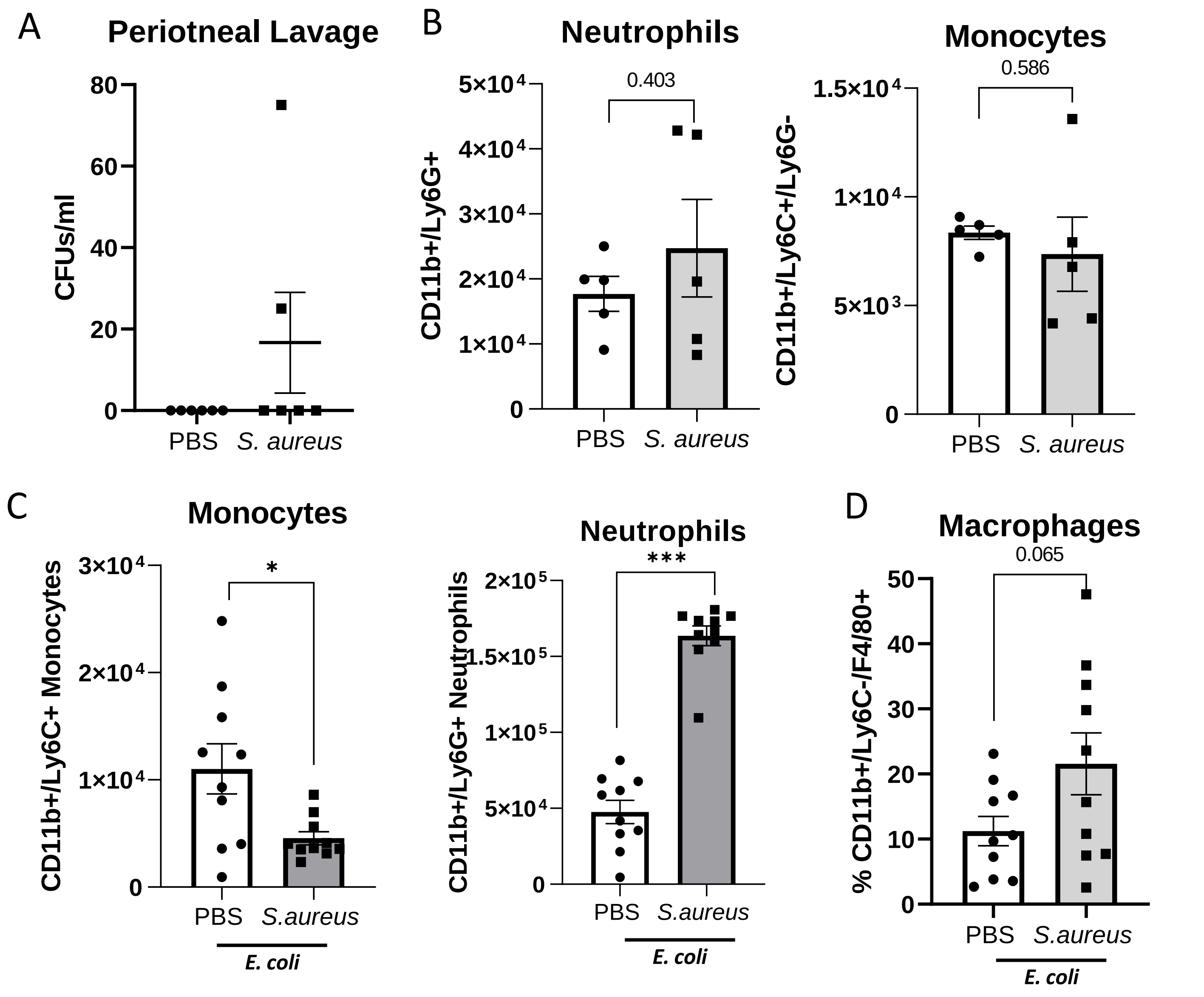

Figure S3. Resident monocyte and neutrophil populations are not elevated in the peritoneal cavity at day 21 post exposure to *S. aureus*.

Groups of C57/Bl6 mice were exposed to *S. aureus* or PBS through i.p injection (5x10^8 cfus/ml) on day 0, 7 and 14. Mice were allowed to rest until day 21 upon which the peritoneal cavity was lavaged. On day 21 post final *S. aureus* exposure, peritoneal lavage was collected to assess bacterial burden. Peritoneal lavage was plated on TSA and allowed to grow overnight and total bacterial burden assessed (A). Proportions of Ly6G+CD11b+ neutrophils and Ly6G-/CD11b+/Ly6C+ monocytes were analysed by flow cytometry (B). Statistical significance was measured by unpaired t-test. On day 21 post final *S. aureus* exposure, mice were challenged with 1.5x10^{8}cfus/ml *E. coli.* 3hr post *E. coli* challenge peritoneal lavage was collected, and macrophage proportions (C) and neutrophil and monocyte absolute numbers (D) were assessed by flow cytometry. Results expressed as absolute cell number, % of cells or CFUs per ml +/- SEM for n=5-10 individual mice.

Figure S4. Gating strategy for myeloid cells in the peritoneal cavity

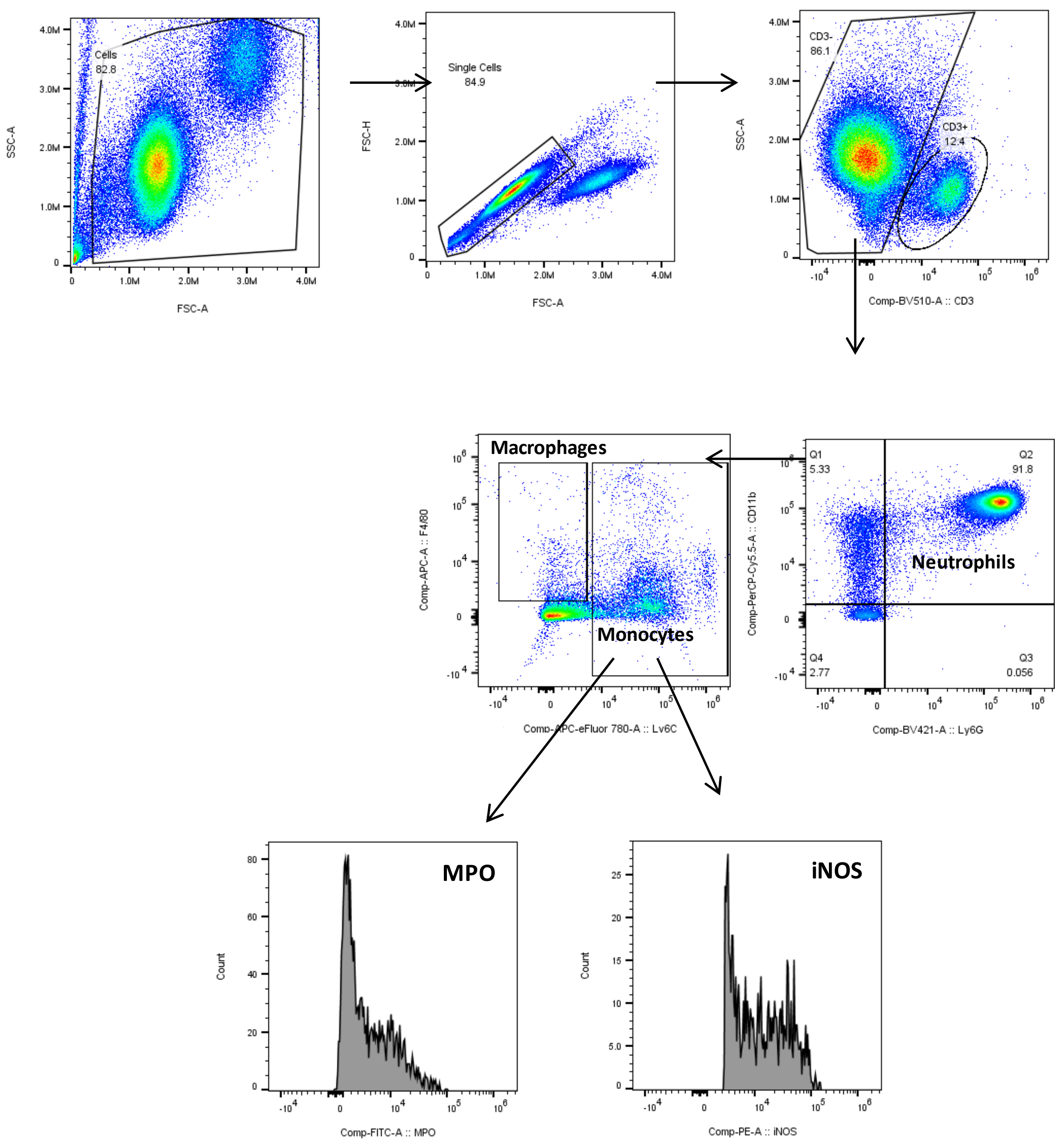

Figure S4. Gating strategy for myeloid cells in the peritoneal cavity.

Groups of C57/Bl6 mice were exposed to *S. aureus* through i.p injection ($5x10^8$ cfus/ml) on day 0, 7 and 14. Mice were allowed to rest for 21 days upon which mice were rechallenged i.p with *E.coli* ($1.5x10^7$ cfus/ml). Peritoneal lavage was collected at 3hr post *E .coli* challenge and cells were stained with fluorochrome-conjugated antibodies against CD3 (BV510), Ly6G (BV421), CD11b (PerCp-Cy5.5), F4/80 (APC), Ly6C (APC-eFlour780), iNOS (PE) and MPO (FITC). Cell debris and doublet populations were gated out before T-cell populations were gated on based on CD3 expression. Neutrophils were delineated with CD11b+ and Ly6G+ markers. Macrophages were delineated with CD11b+ and F4/80 markers. Monocytes were delineated by Ly6G-, CB11b+ and Ly6C+. MPO and iNOS expression was quantified by median fluorescence intensity.

Figure S5. *S. aureus* exposure in mice offers protection against subsequent *E. coli* challenge in absence of T-cells.

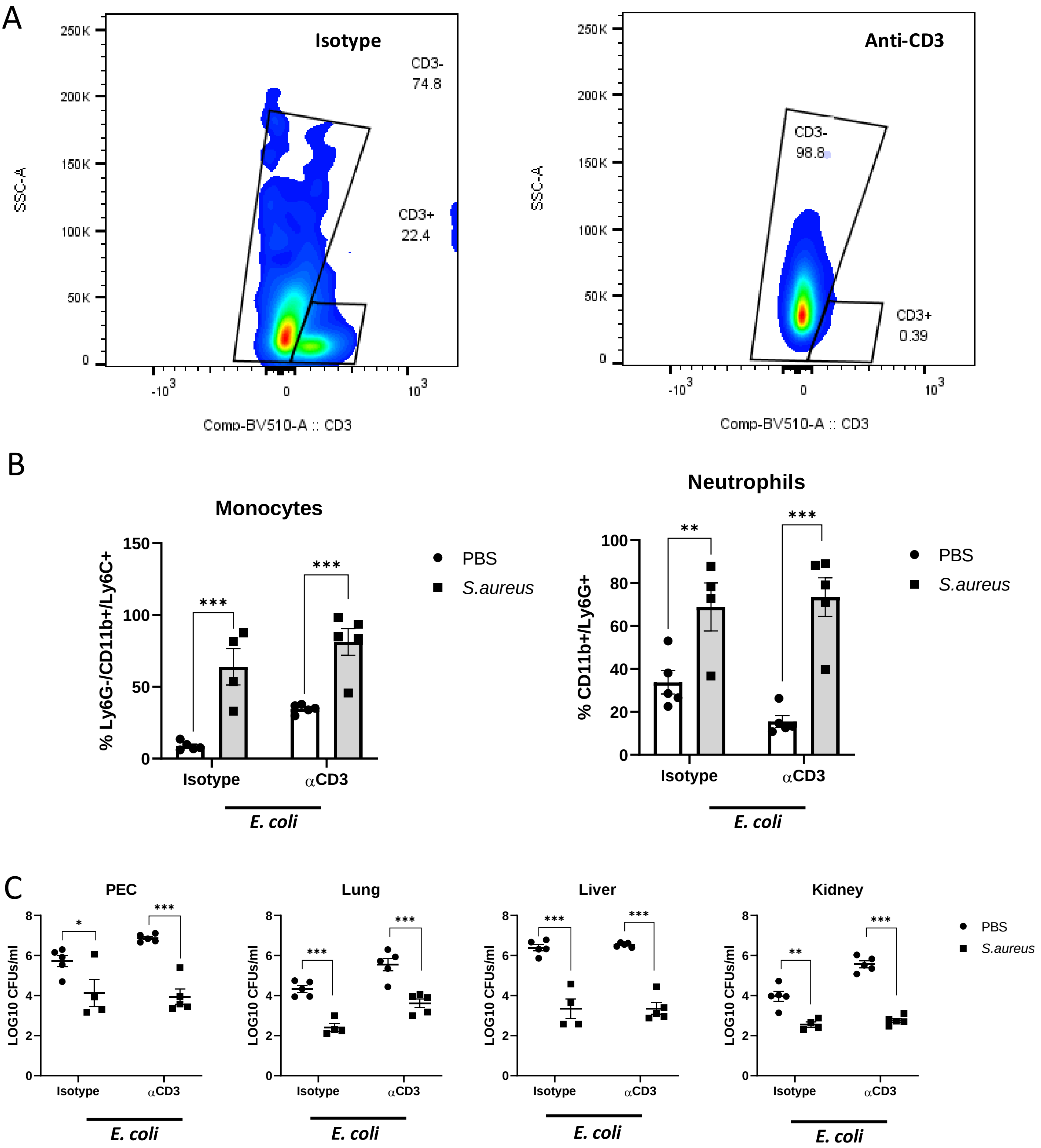

Figure S5. *S. aureus* exposure in mice offers protection against subsequent *E. coli* challenge in absence of T-cells.

Groups of C57/Bl6 mice were exposed to *S. aureus* through i.p injection ($5x10^8$ cfus/ml) on day 0, 7 and 14. Mice were allowed to rest for 21 days. On day 34, mice received anti-CD3 antibody (200µg/mouse) or IgG Isotype control (200µg/mouse). On day 35 mice were rechallenged i.p with *E. coli* ($1.5x10^7$ cfus/ml). Depletion of CD3+ cells was confirmed by flow cytometry (A). Proportions of Ly6G+CD11b+ neutrophils and Ly6G-/CD11b+/Ly6C+ monocytes were analysed by flow cytometry at 3hr post *E. coli* challenge (B). At 3h post *E. coli* challenge peritoneal cavity was lavaged and kidneys, lungs, livers were excised and homogenised. Lavage and homogenates were plated on TSA and allowed to grow overnight and total bacterial burden assessed (C). Results expressed as % of cells, LOG10 CFUs/ml +/-SEM for n=5 individual mice. Statistical significance measured by sidak test, $p < 0.05$ *, $p < 0.01$ **, $p < 0.0001$ ***.

Figure S6 Gating strategy for bone marrow phenotyping

A

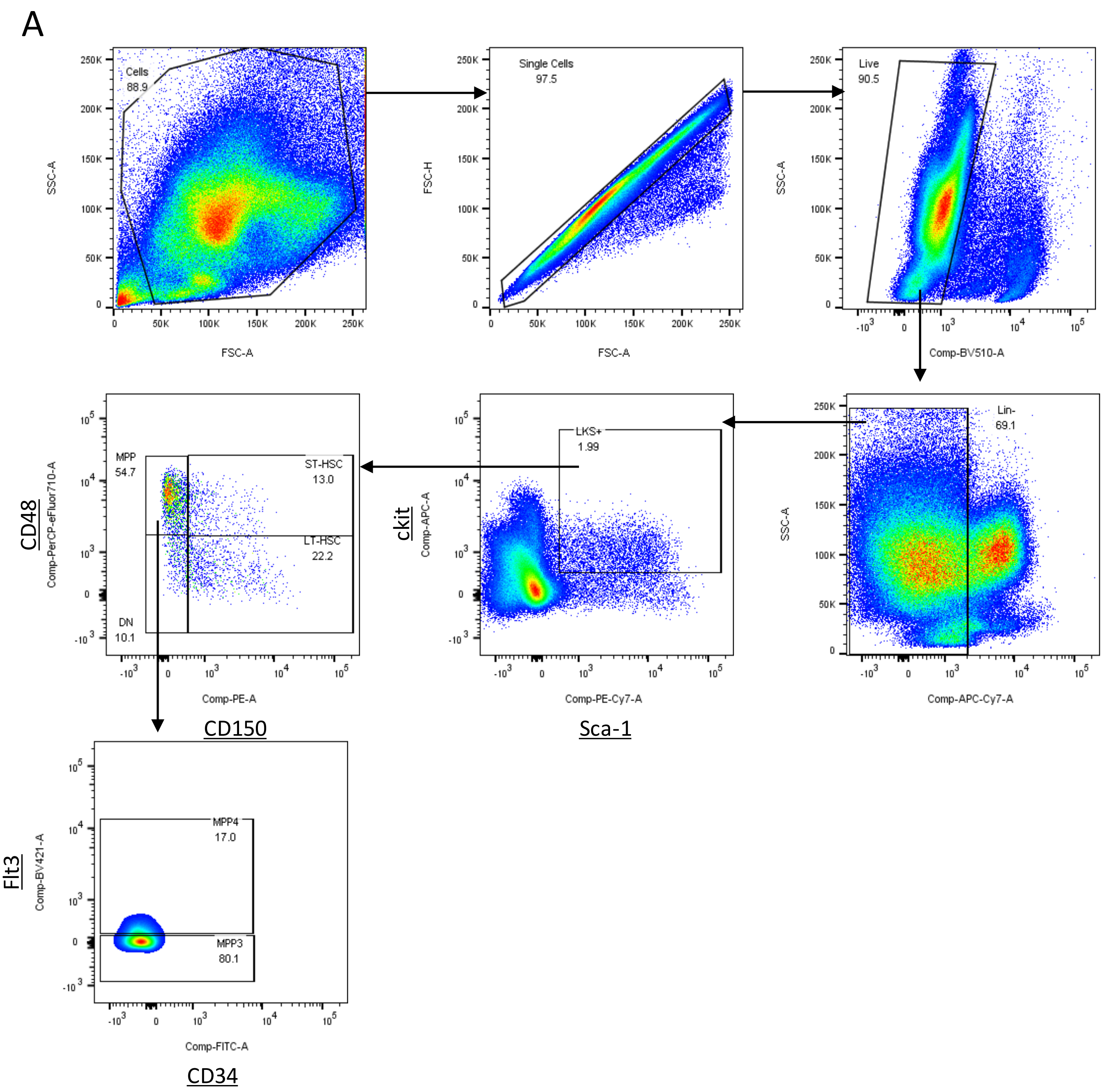

B

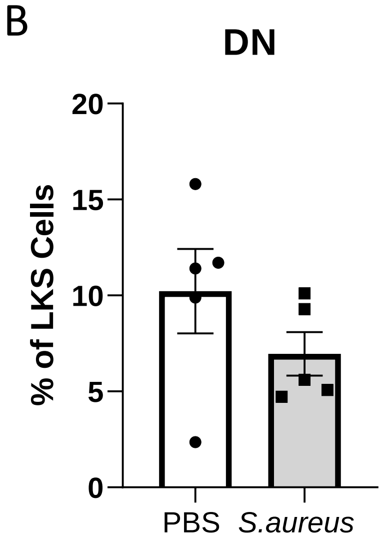

Figure S6. Gating strategy for myeloid cells in the peritoneal cavity

Groups of C57/Bl6 mice were exposed to *S. aureus* through i.p injection ($5x10^8$ cfus) on day 0, 7 and 14. Mice were allowed to rest for 21 days upon which bone marrow cells were isolated for phenotypic analysis by flow cytometry. Cells were stained with Fixable Viability e506, followed by Ly6C, Ly6G, CD8a, TER119, CD5, B220 (all APC-Cy7), Sca-1 (PeCy7), CD127 (BV711), CD34 (FITC), CD48 (PerCPef710), CD150 (PE), Flt3 (BV421), cKit (APC), and CD16/32 (AF700). Cell debris and doublet populations were gated out before dead cells were gated out. Lineage positive (Ly6C, Ly6G, CD8a, TER119, CD5, B220) cell were gated out. LKS cells ($Lineage^-cKit^+Sca1^+$) were delineated by expression of cKit and Sca-1. LKS cells were further delineated into LT-HSC (LKS+CD48-CD150+) ST-HSC ($LKS^+CD48^+CD150^+$) and MPP cells by expression of CD48 and CD150. MPP cells were subdivided into MPP3 (LKS+CD48+CD150-Flt3+) and MPP4 (LKS+CD48+CD150-Flt3-) cells by expression of Flt3 and CD34. Proportion of double negative cells (CD48-CD150-). Results expressed as % of cells +/-SEM for n=5 individual mice.

Figure S7 Gating strategy for myeloid cells in the bone marrow and circulation

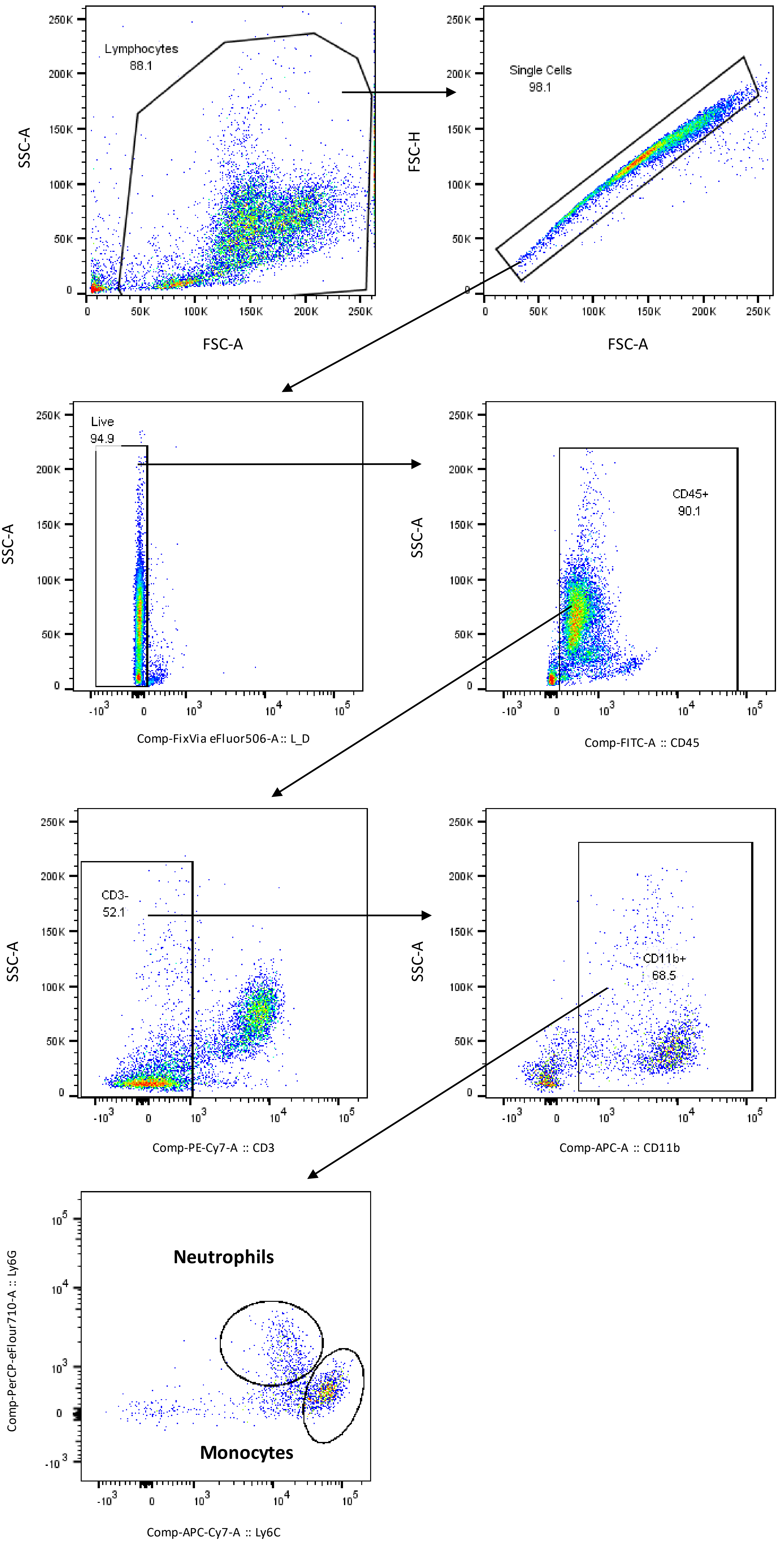

Figure S7. Gating strategy for myeloid cells in the peritoneal cavity

Groups of C57/Bl6 mice were exposed to *S. aureus* through i.p injection (5x10^8 cfus/ml) on day 0, 7 and 14. Mice were allowed to rest for 21 days. Bone marrow cells were isolated from the femur of the mouse and blood was collected by cardiac puncture. Red blood cells were lysed for 30 minutes on ice use ACK lysis buffer (Sigma). Cells were stained with antibodies against CD45(FITC), CD3 (PeCy7), CD11b (APC), Ly6C (APC-Cy7) and Ly6G (PerCPeflour710) as well as Fixable Viability e506. Cells were gated to remove debris, doublets and dead cells. Cells were gated to remove CD45- cells and CD3+ cells. Cells were then gated to identify the CD11b+LyC6+ Monocytes and CD11b+Ly6G+ Neutrophils.

Figure S8. *S. aureus* exposure enhances peritoneal macrophage effector functions

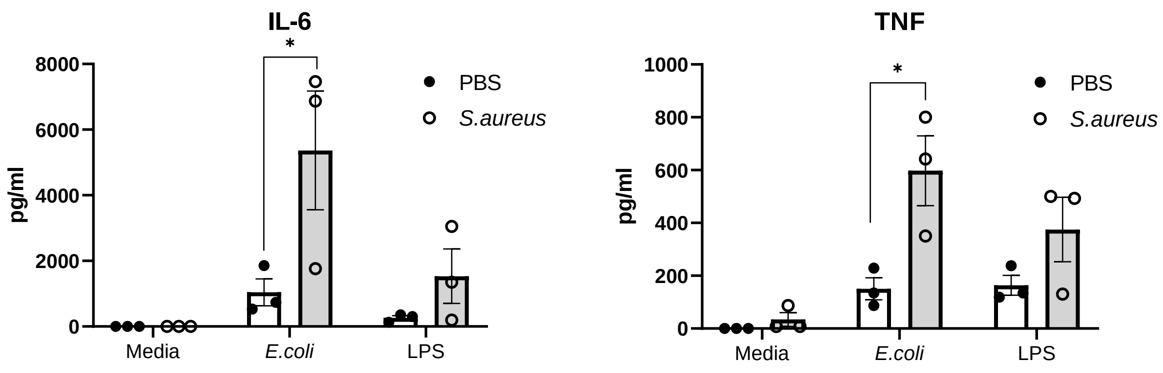

Figure S8. *S. aureus* exposure enhances peritoneal macrophage effector functions

Groups of C57/Bl6 mice were exposed to *S. aureus* through i.p injection (5x10^8 cfus/ml) on day 0, 7 and 14. Mice were allowed to rest for 21 days upon which the peritoneal cavity was lavaged to isolate resident peritoneal macrophages. Peritoneal macrophages were stimulated in vitro with LPS (10ng/ml) and *E. coli* (MOI 100) and IL-6 and TNF production measured by ELISA at 24hr post stimulation. Results expressed as mean pg/ml +/-SEM for n=3 individual mice. Statistical significance measured by unpaired t-test, $p < 0.05$ *.

Figure S9. *S. aureus* exposure enhances peritoneal macrophage effector functions

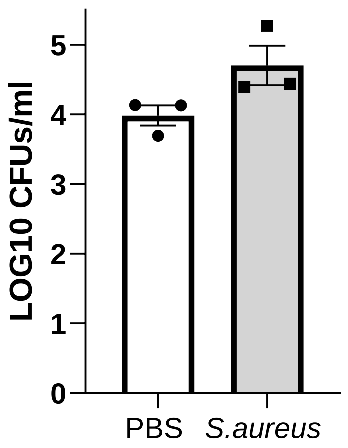

Figure S9. *S. aureus* exposure does not impact *E. coli* intracellular survival in macrophages Groups of C57/Bl6 mice were exposed to *S. aureus* through i.p injection (5x10^8 cfus/ml) on day 0, 7 and 14. Mice were allowed to rest for 21 days upon which BMDMs were infected with *E. coli* at a multiplicity of infection (MOI) of 100 for 1 h before gentamicin treatment (200 µg/ml). for a further 1 h After this time, gentamicin containing media was removed and replaced with antibiotic free cDMEM. At 24hr post challenge cells were lysed with PBS containing Triton X-100. Lysates were plated on TSA and allowed to grow overnight upon which cfus were enumerated. Results expression as mean CFU/ml. +/-SEM for BMDMs isolated from n=3 independent animals per group.
